# Supplementary material for: Robustness of statistical methods when measure is affected by ceiling and/or floor effect
Source: PLoS One. 2019 Aug 19;14(8):e0220889. doi: 10.1371/journal.pone.0220889 (PMC6699673; doi:10.1371/journal.pone.0220889)
Supplement: S1 Appendix — See the introductory paragraph of section 3 for details. (PDF) [file pone.0220889.s001.pdf]

Supplement to Robustness of statistical methods when measure is affected by ceiling and/or floor effect

Matus Simkovic[1] **and Birgit Träuble**[1]

[1]**Universität zu Köln, Cologne, Germany**

Corresponding author:
Matus Simkovic[1]

Email address: matus.simkovic@uni-koeln.de

ABSTRACT

1 GENERALIZED GAMMA DISTRIBUTION

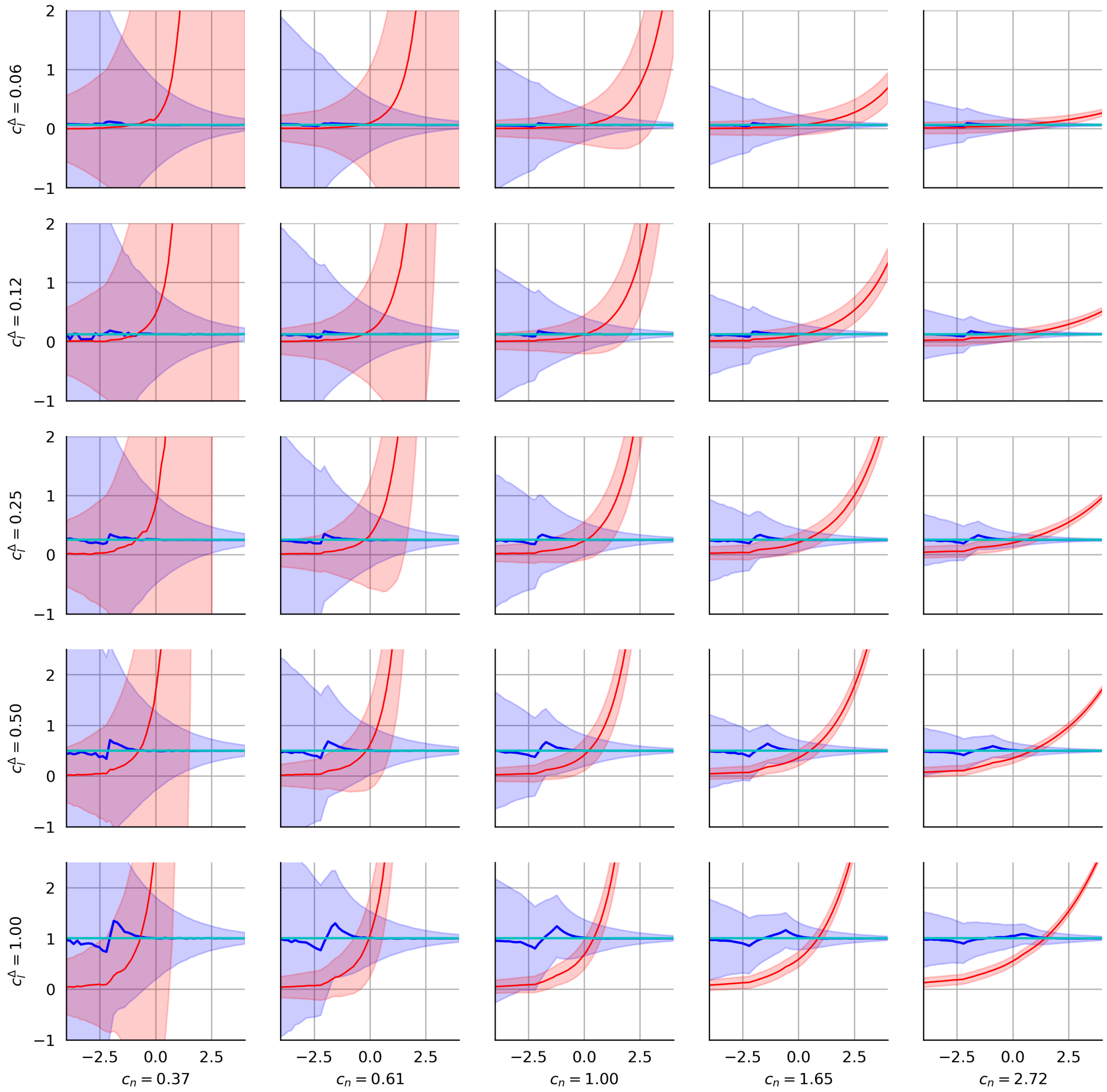

Figure 1. Confidence intervals evaluated with Generalized gamma distribution

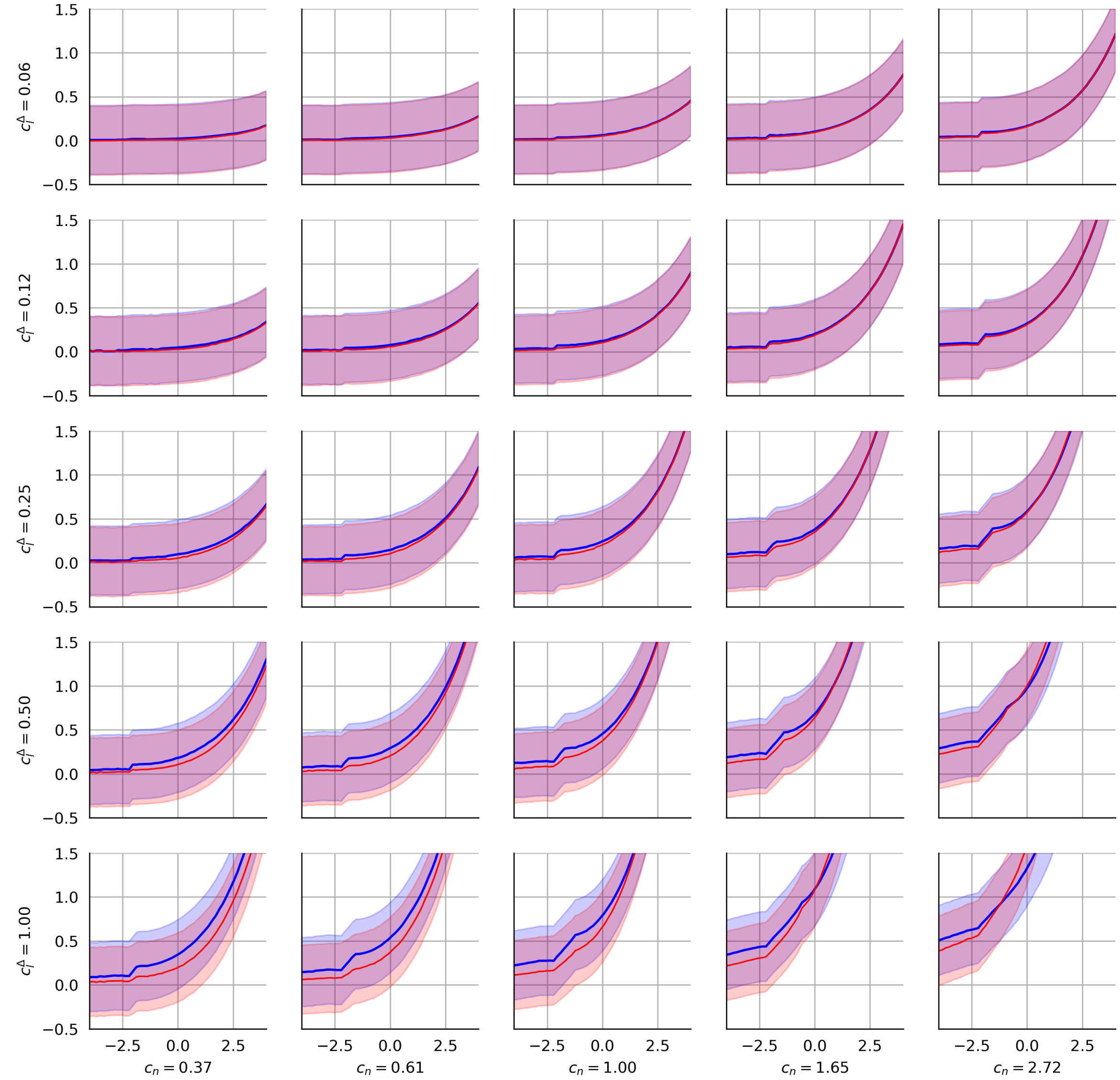

Figure 2. Cohen's d evaluated with Generalized gamma distribution

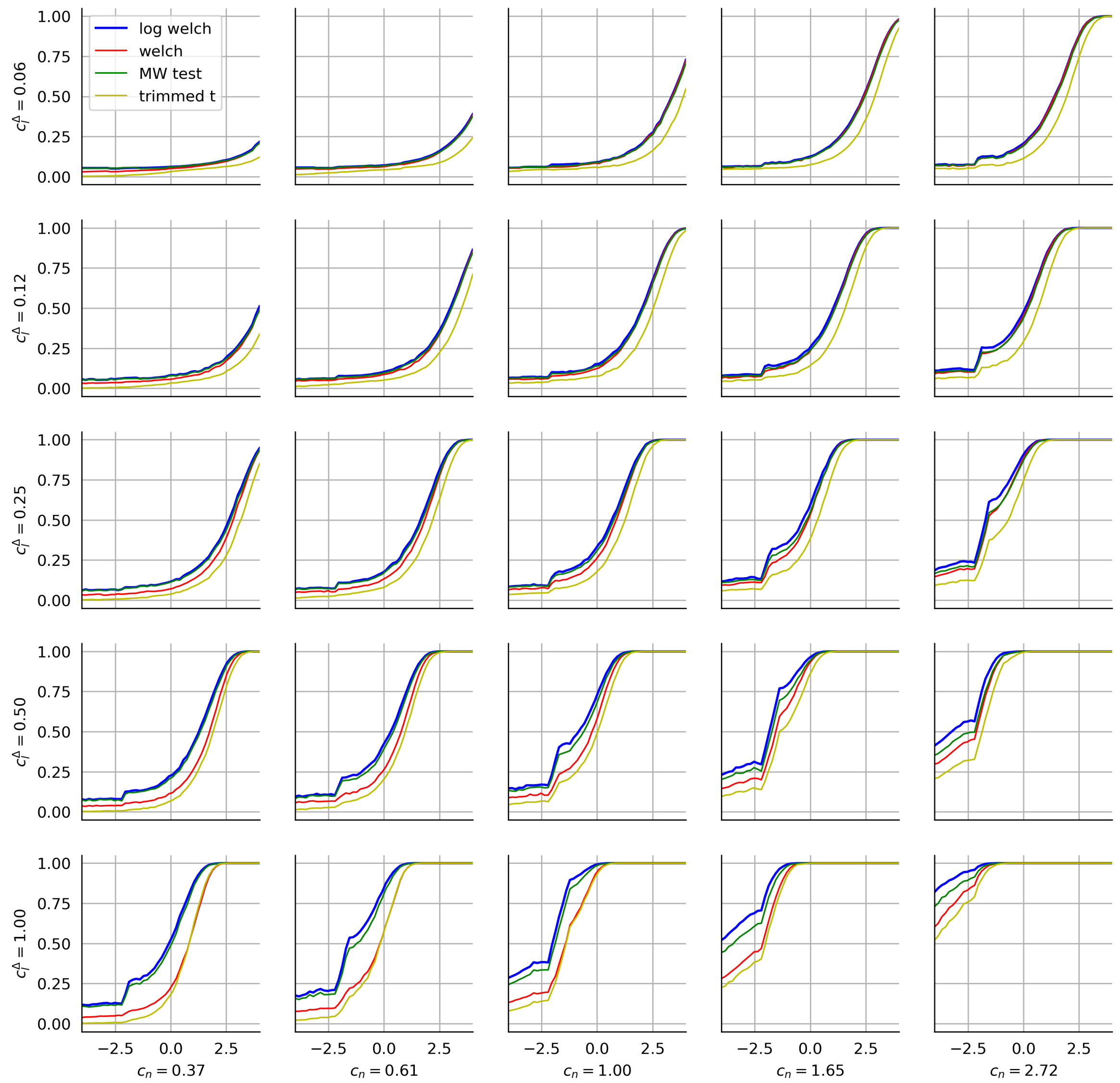

Figure 3. Two-group tests evaluated with Generalized gamma distribution

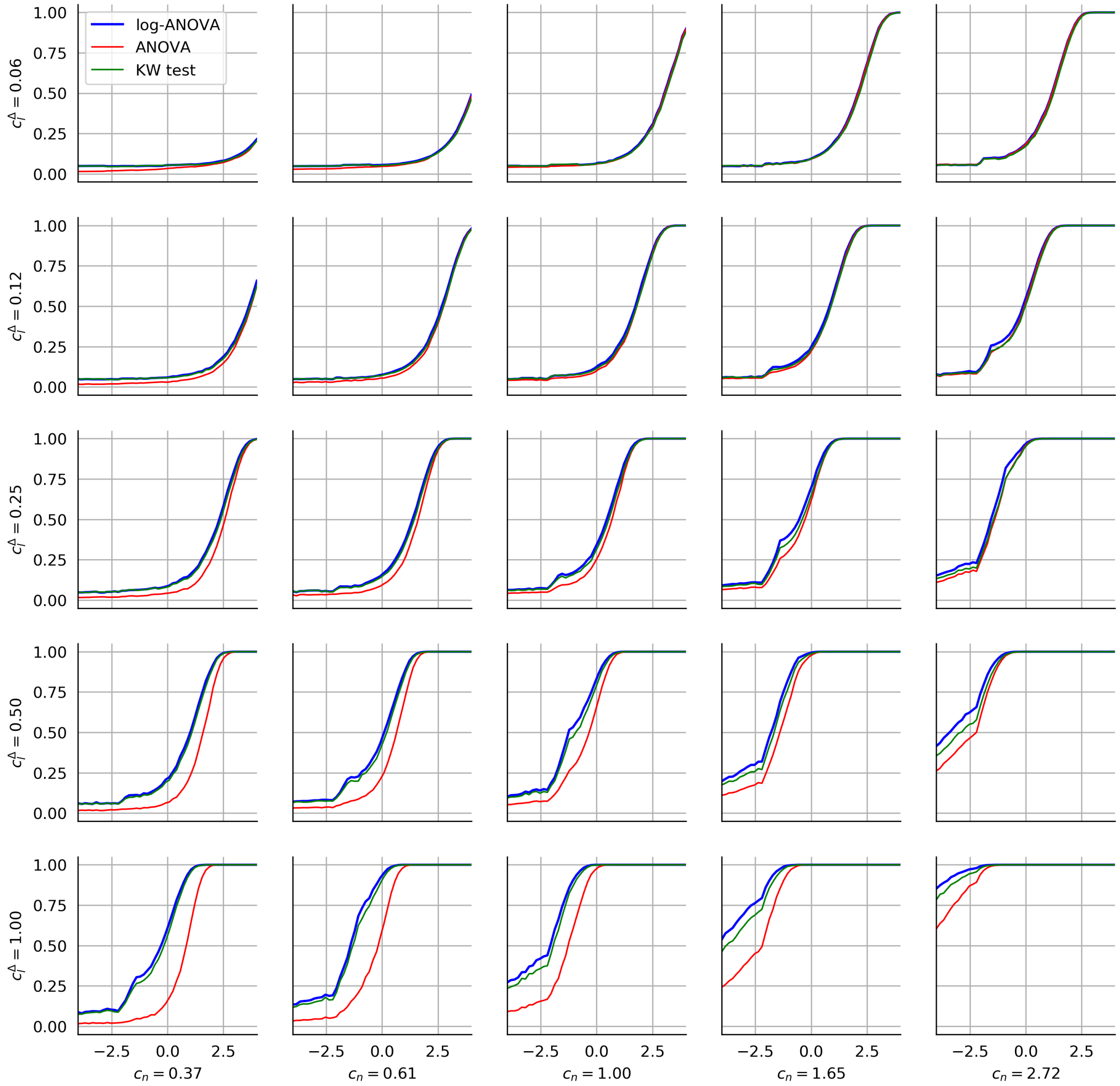

Figure 4. Three-group tests evaluated with Generalized gamma distribution

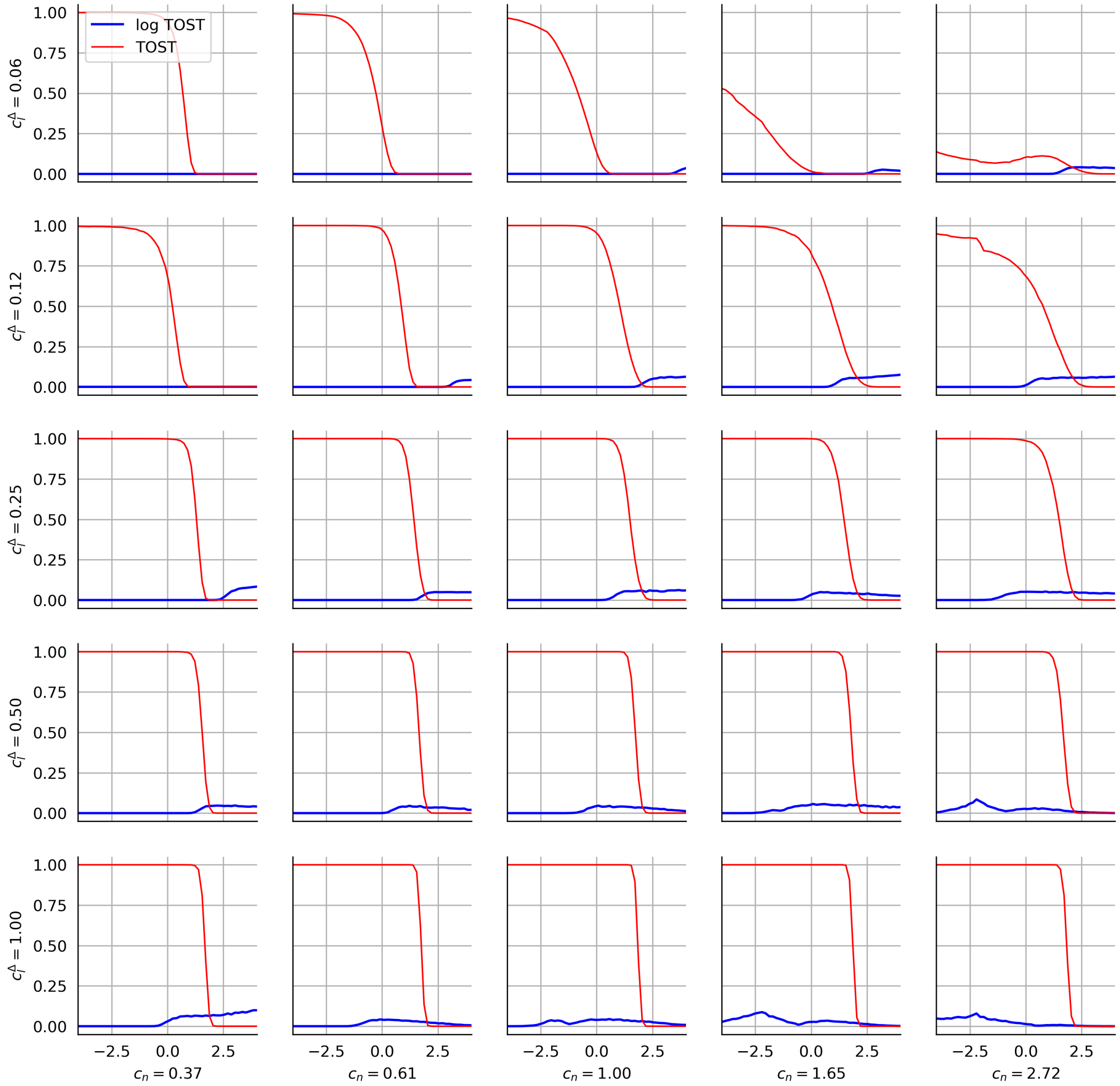

Figure 5. TOST procedure evaluated with Generalized gamma distribution

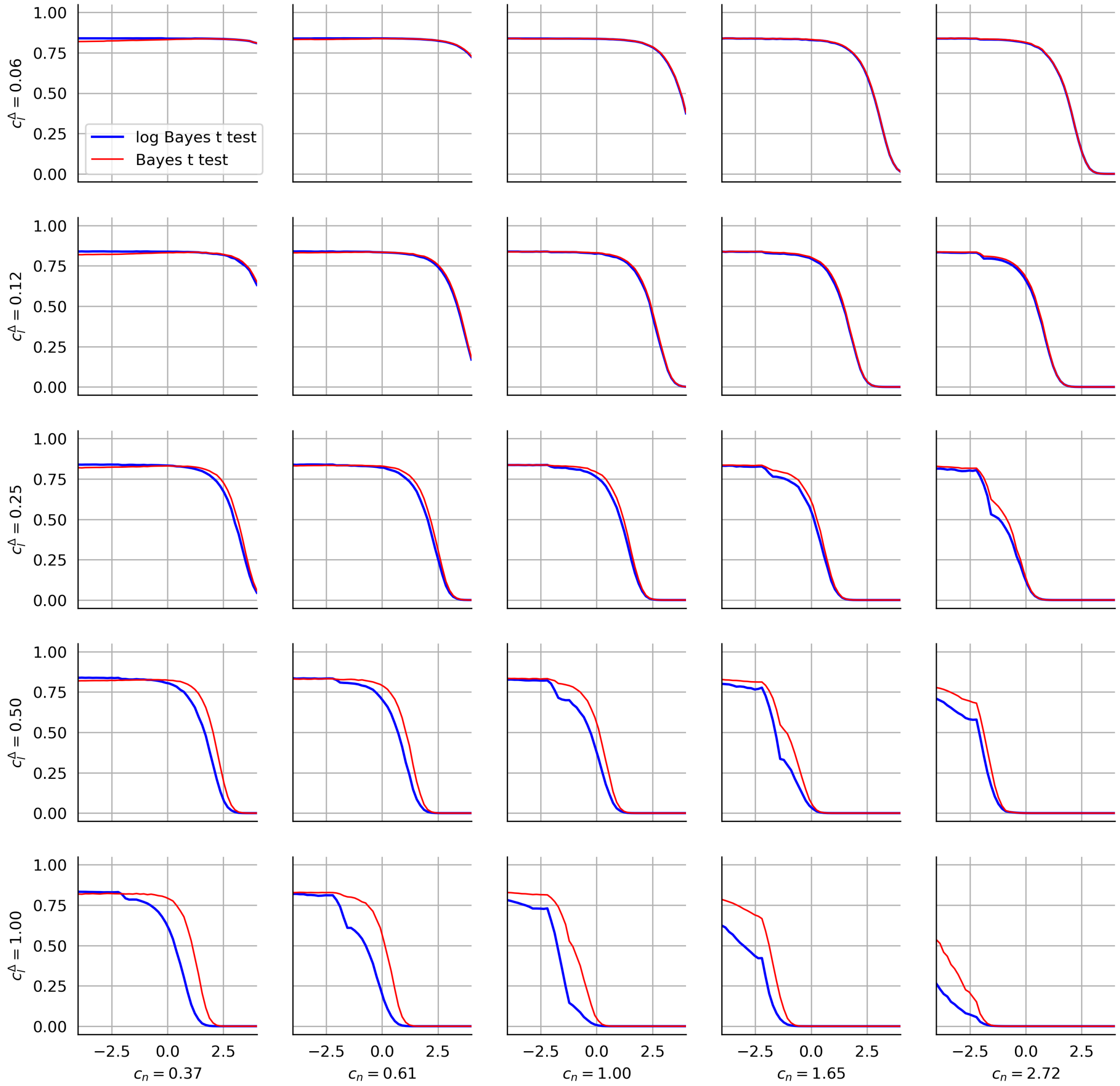

Figure 6. Bayesian t test evaluated with Generalized gamma distribution

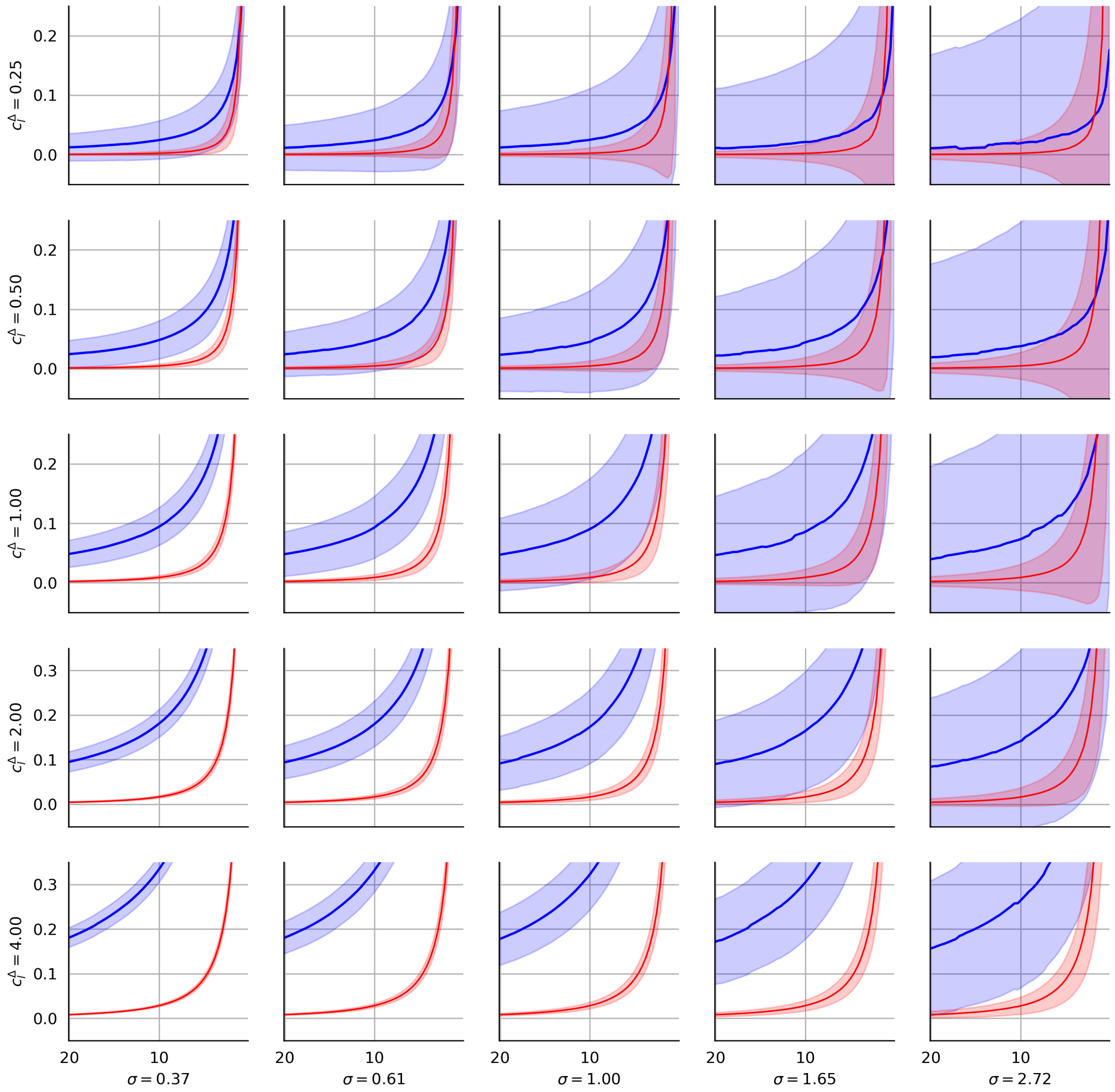

Figure 7. Confidence intervals evaluated with Wald distribution

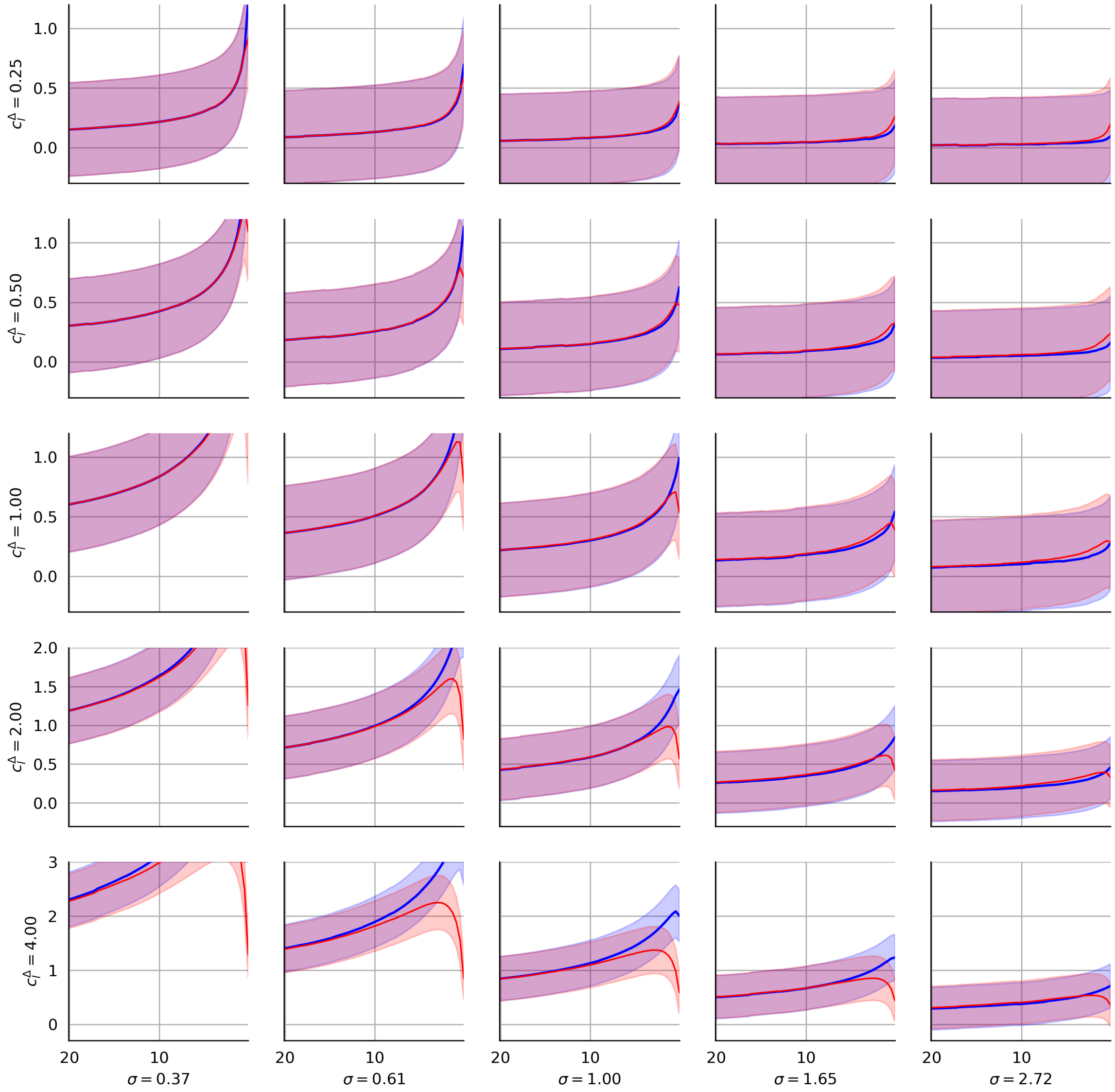

Figure 8. Cohen's d evaluated with Wald distribution

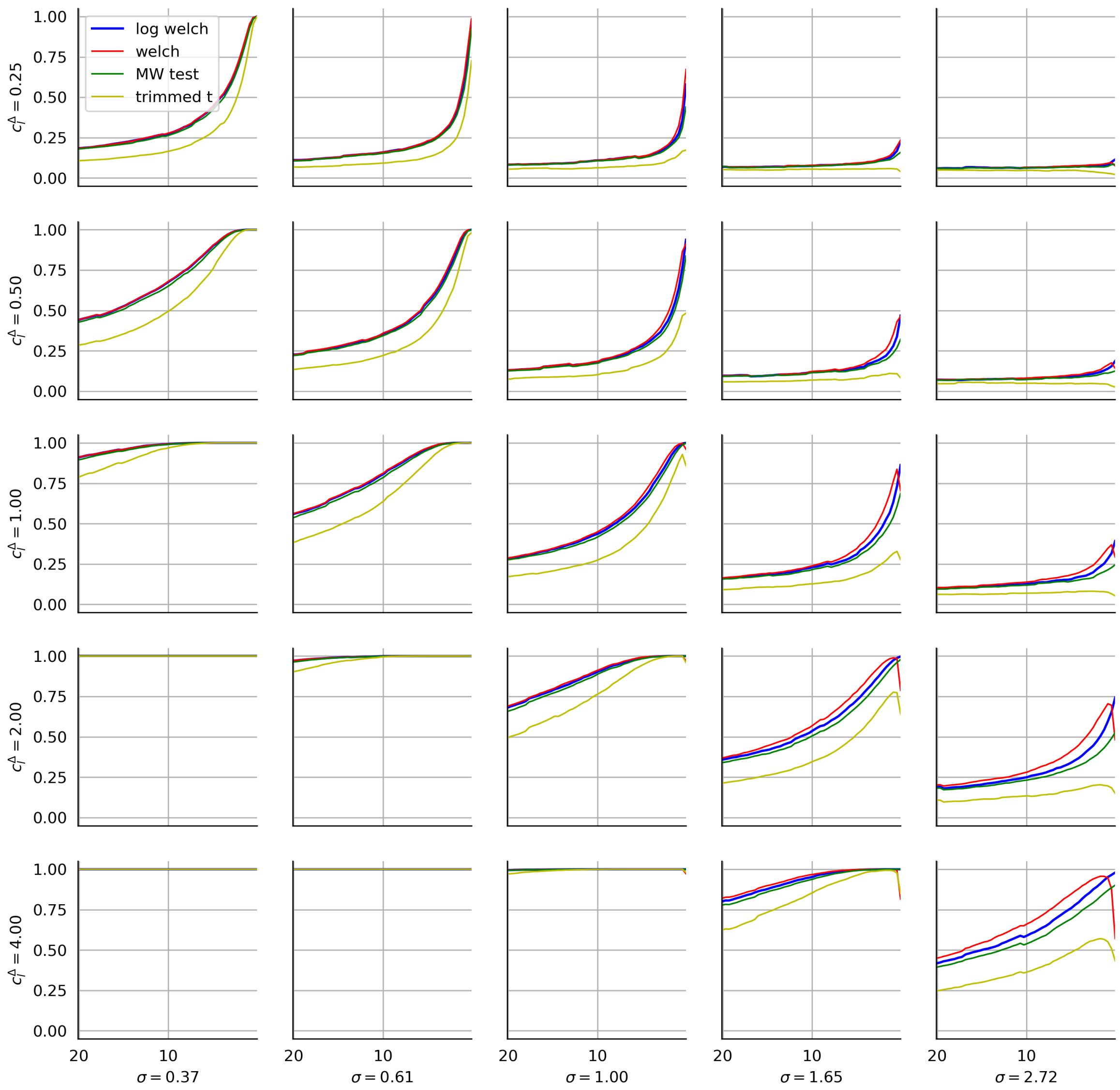

Figure 9. Two-group tests evaluated with Wald distribution

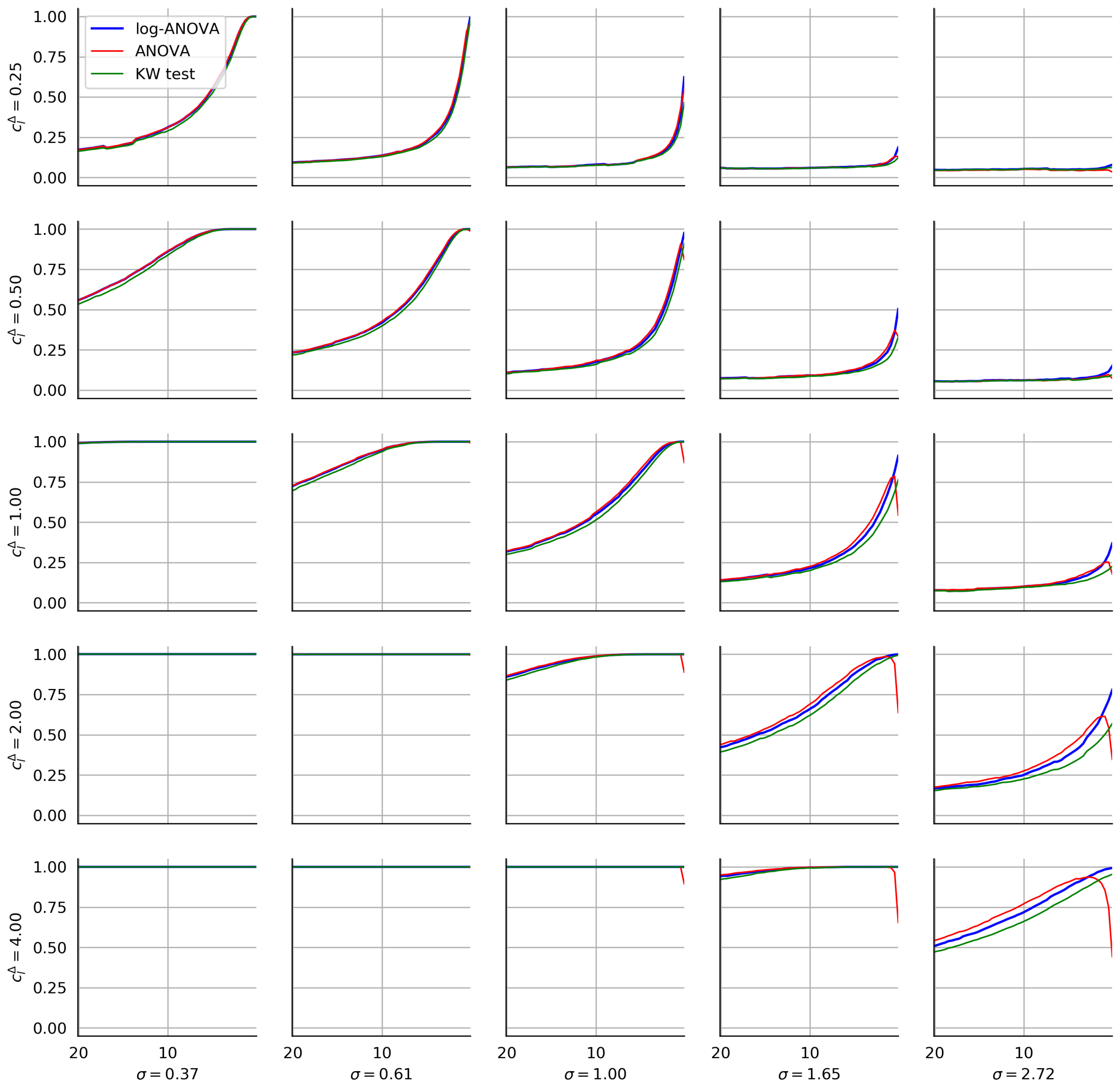

Figure 10. Three-group tests evaluated with Wald distribution

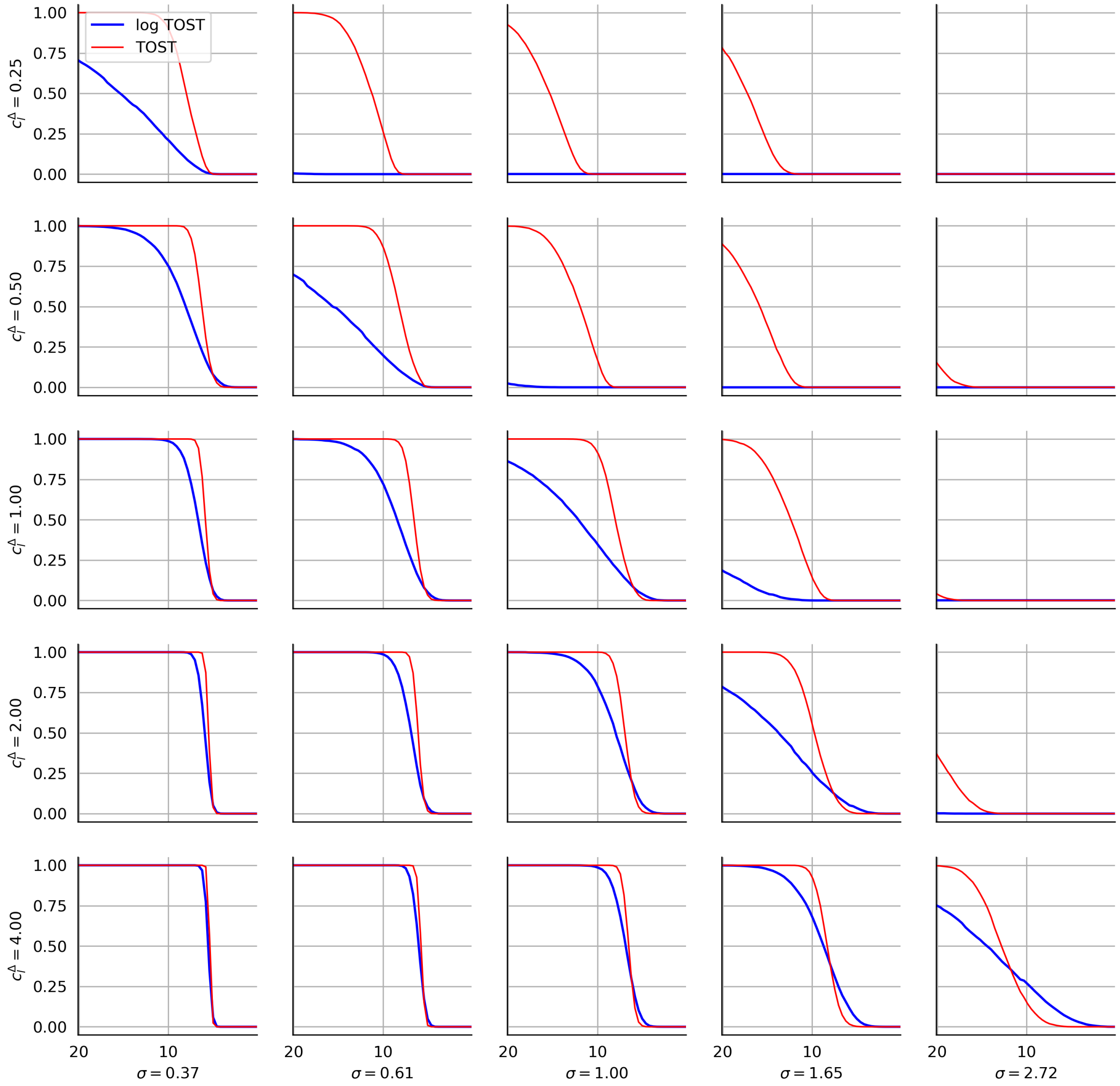

Figure 11. TOST procedure evaluated with Wald distribution

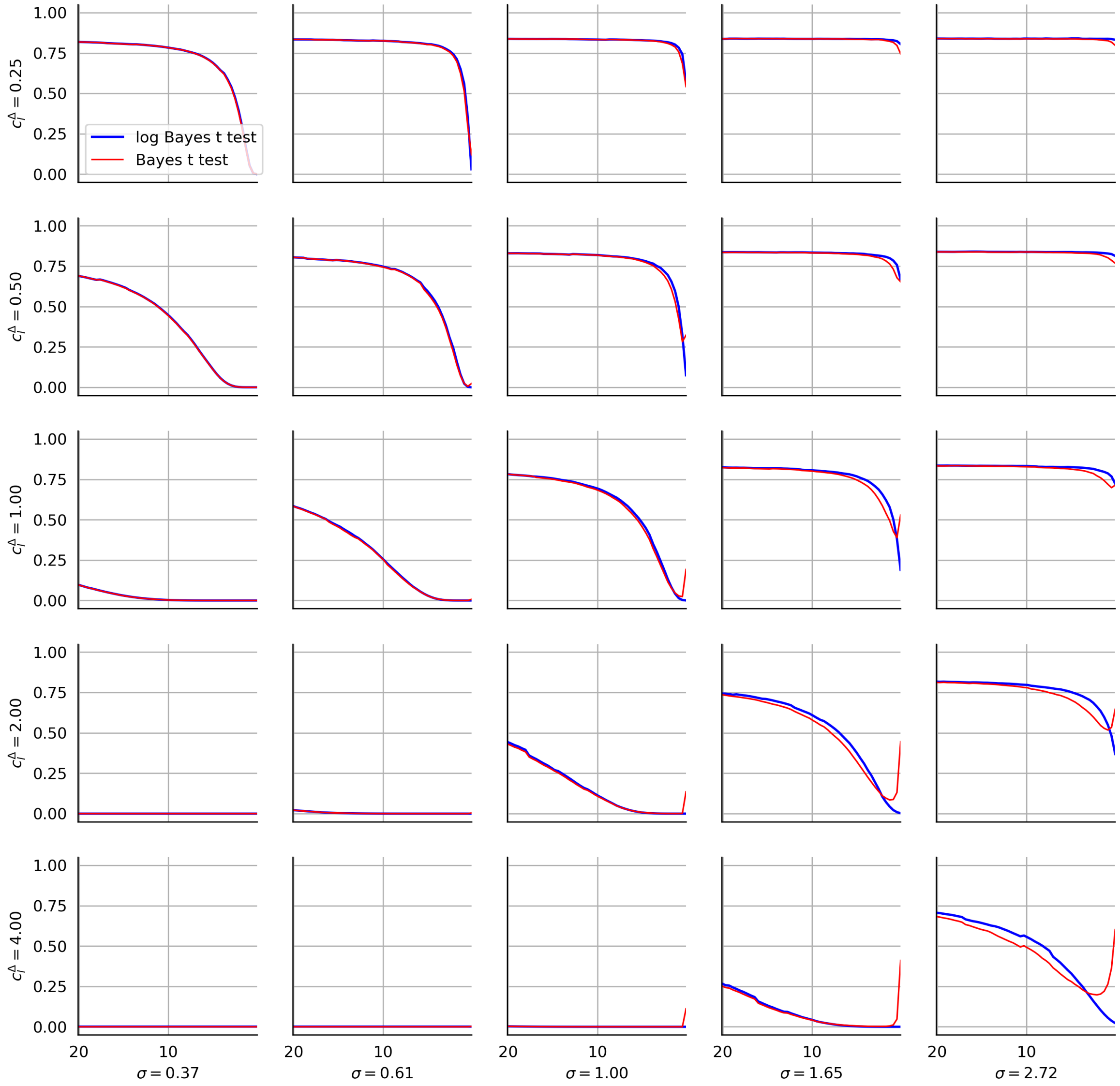

Figure 12. Bayesian t test evaluated with Wald distribution

3 BETA PRIME DISTRIBUTION

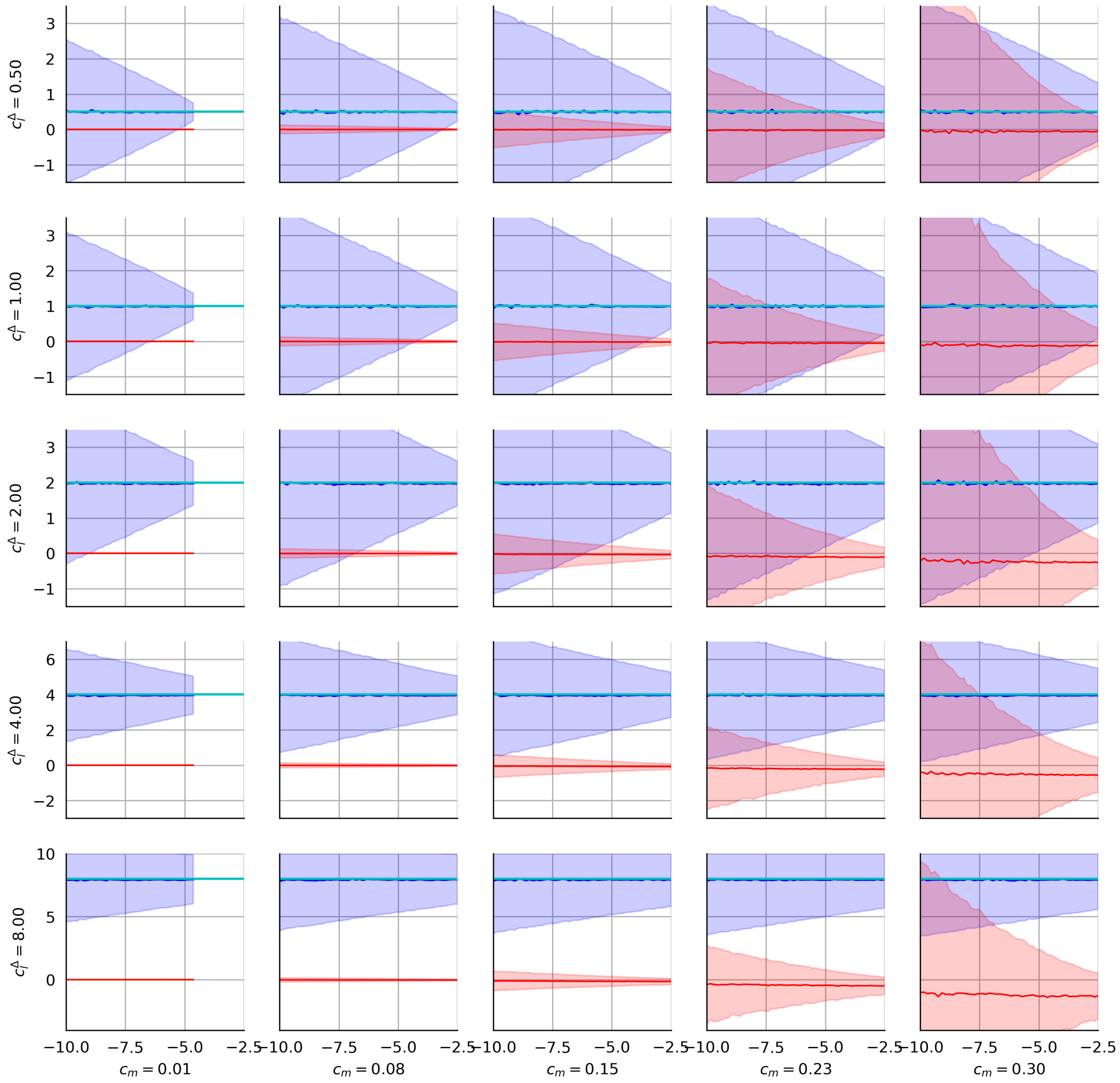

Figure 13. Confidence intervals evaluated with Beta prime distribution

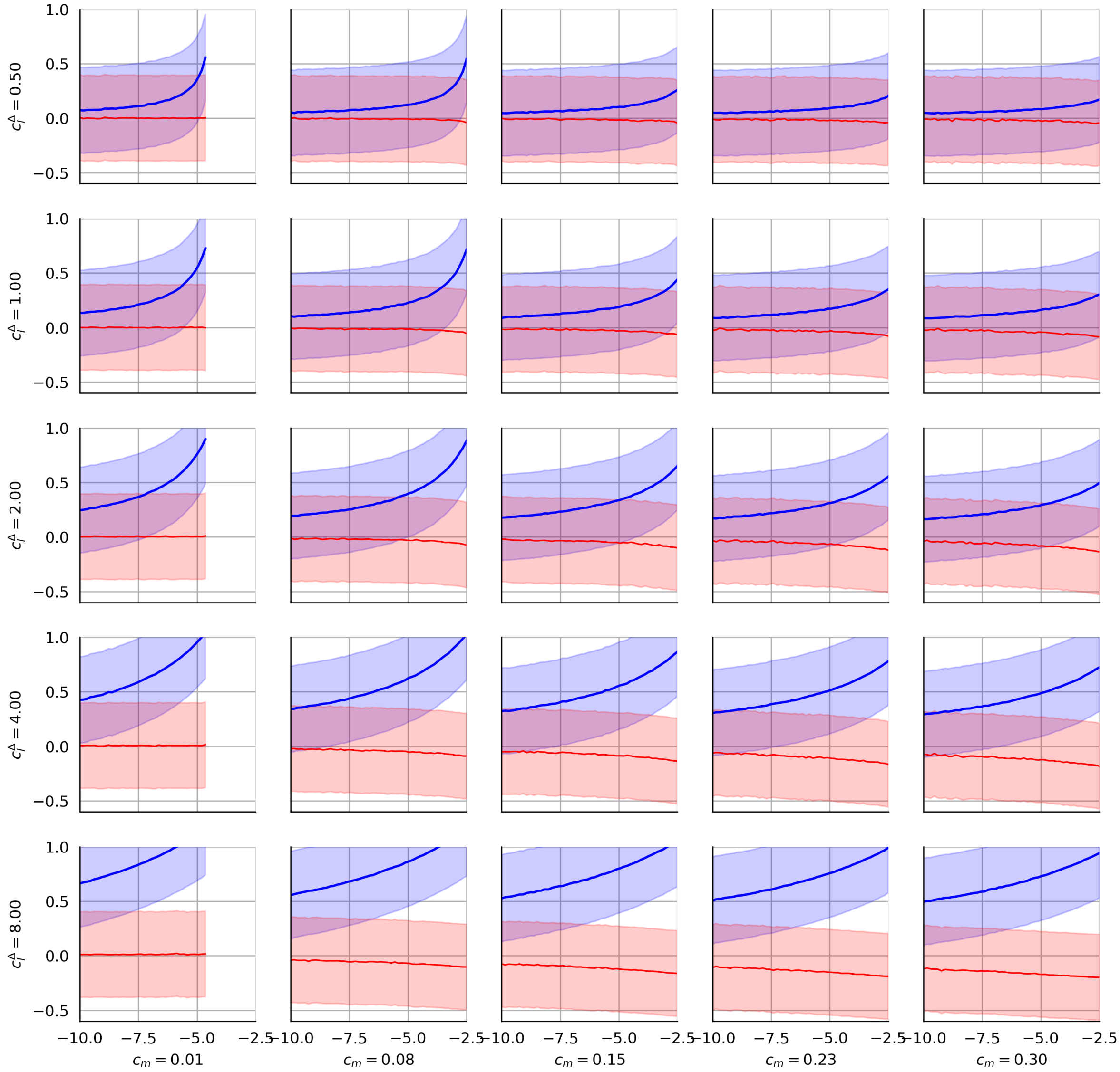

Figure 14. Cohen's d evaluated with Beta prime distribution

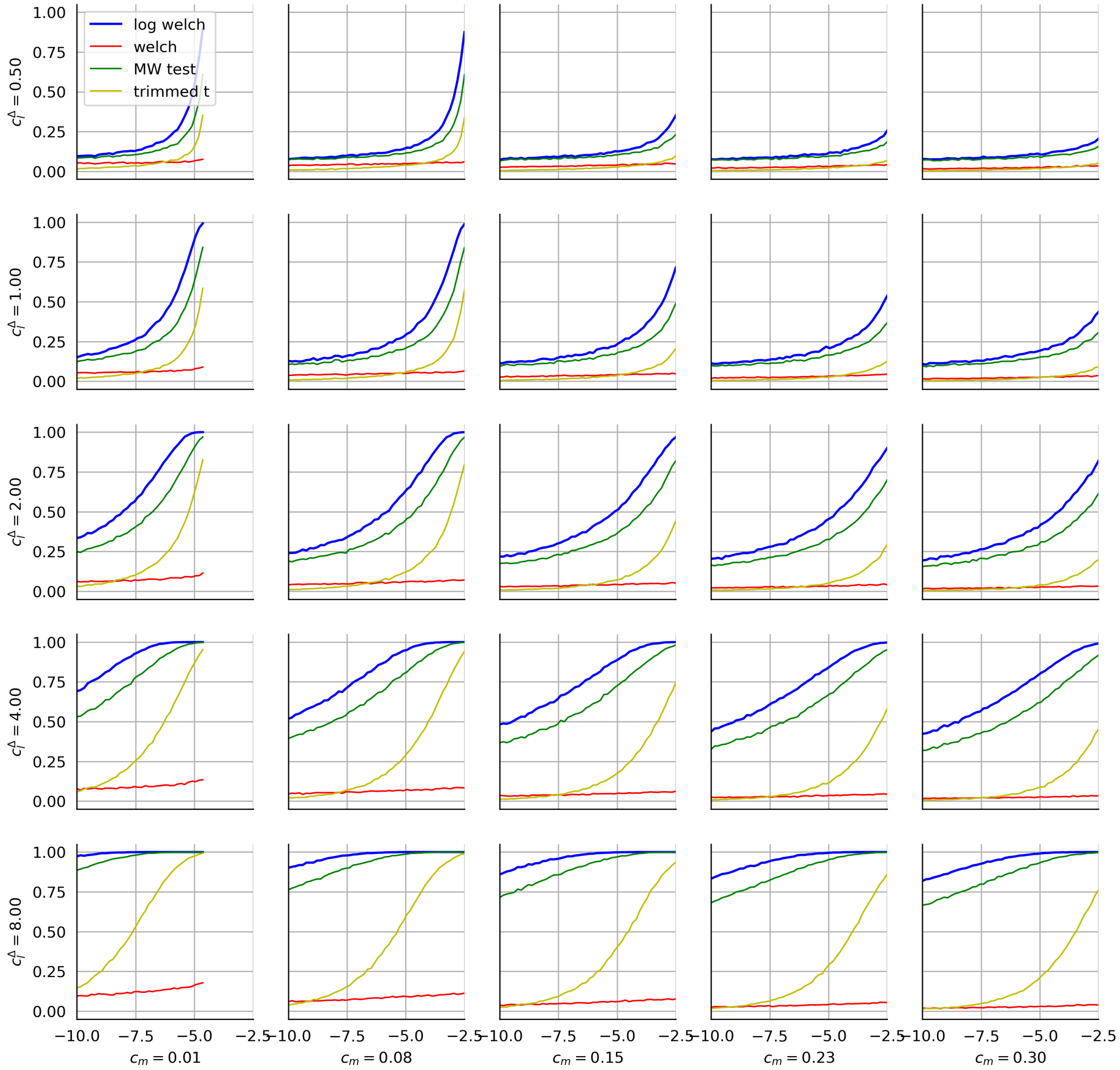

Figure 15. Two-group tests evaluated with Beta prime distribution

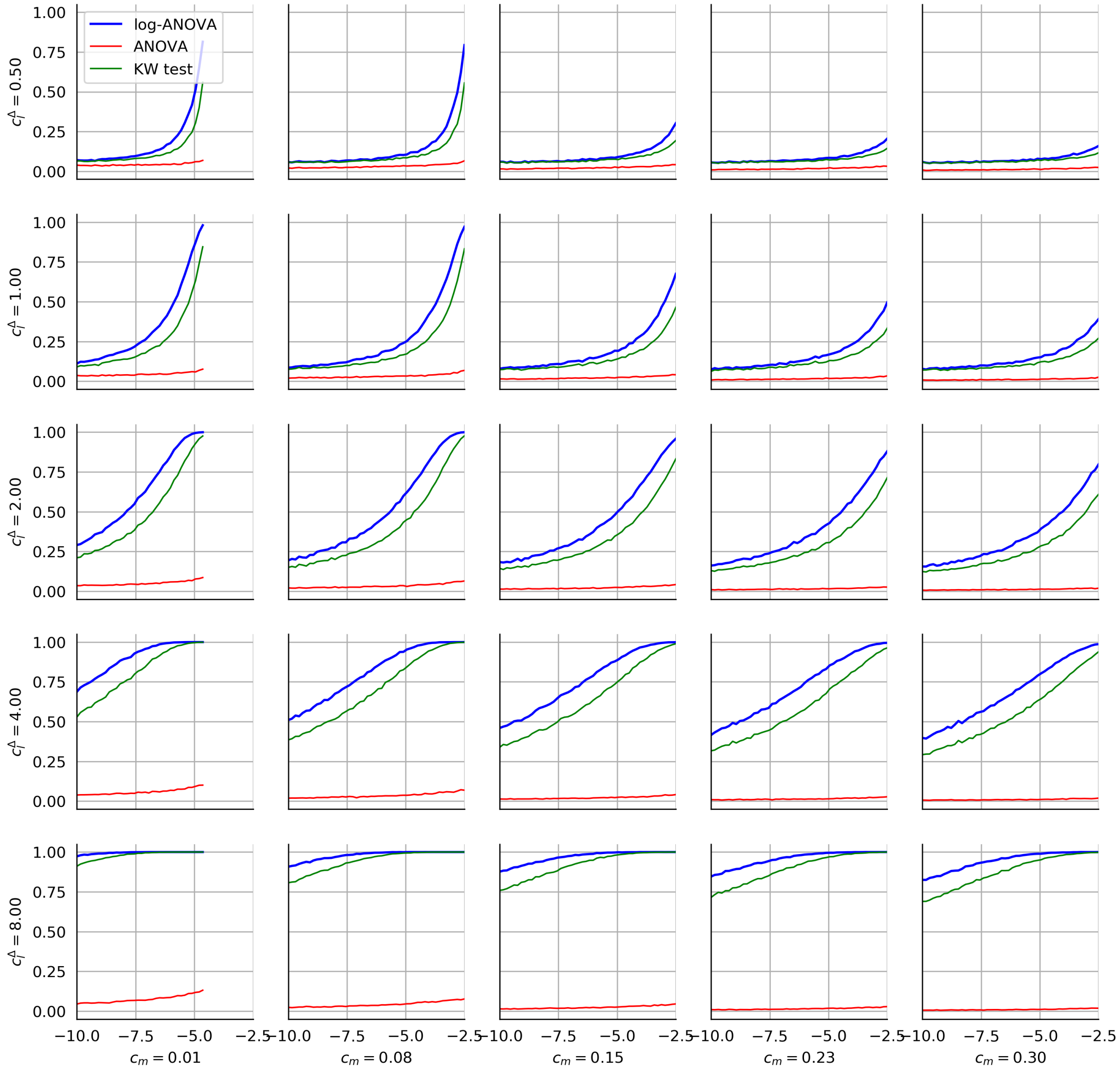

Figure 16. Three-group tests evaluated with Beta prime distribution

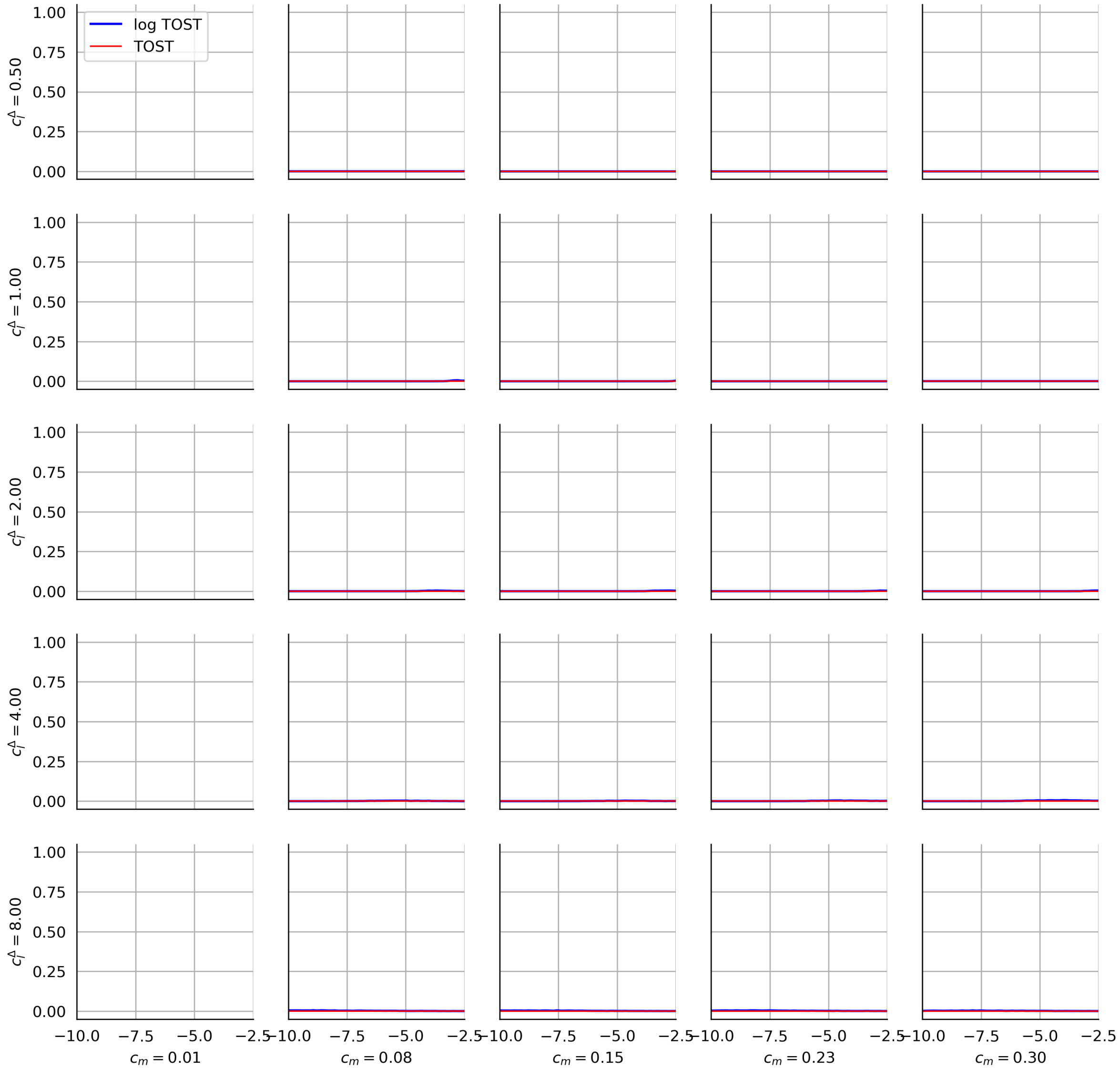

Figure 17. TOST procedure evaluated with Beta prime distribution

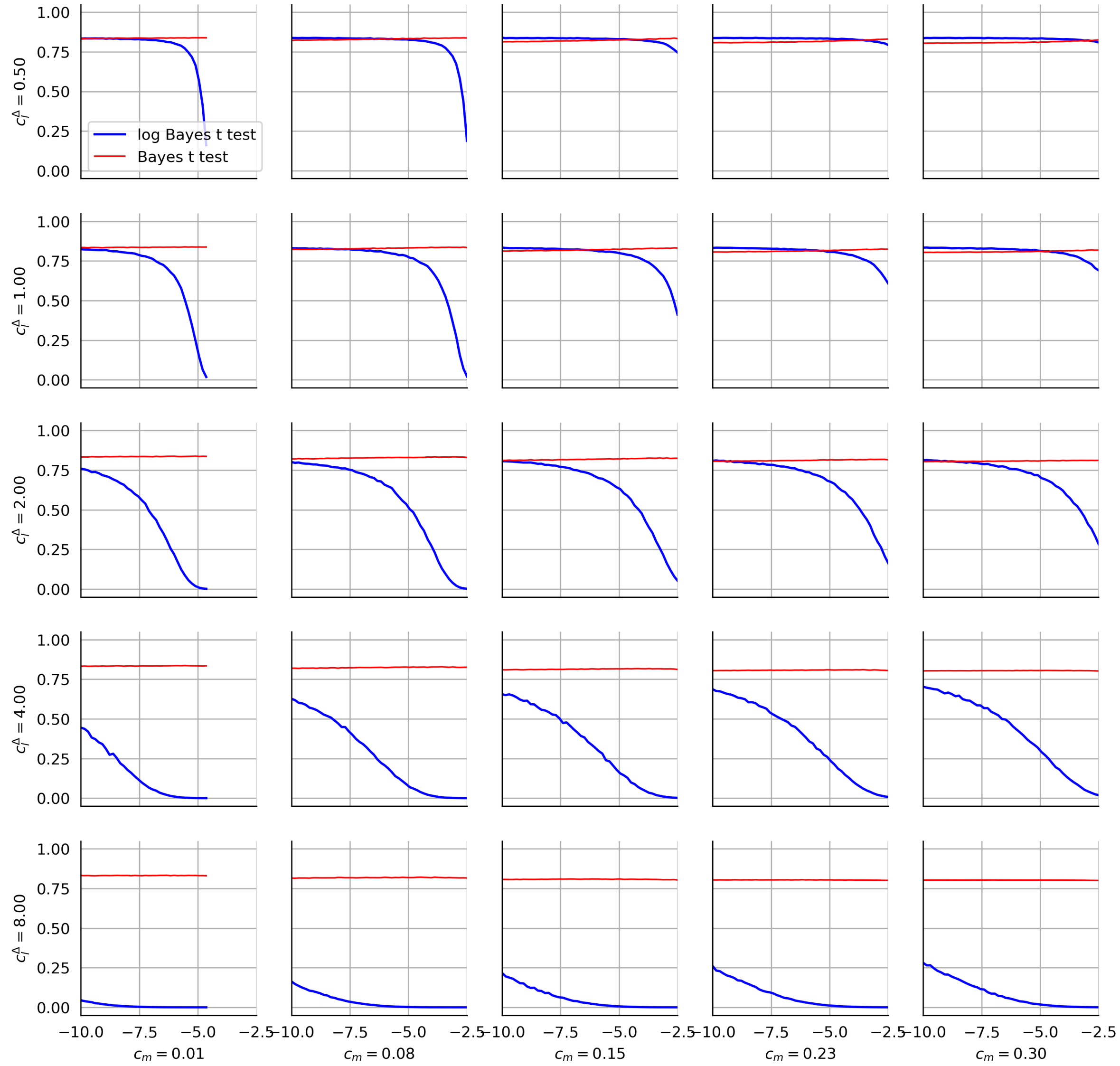

Figure 18. Bayesian t test evaluated with Beta prime distribution

4 BETA DISTRIBUTION

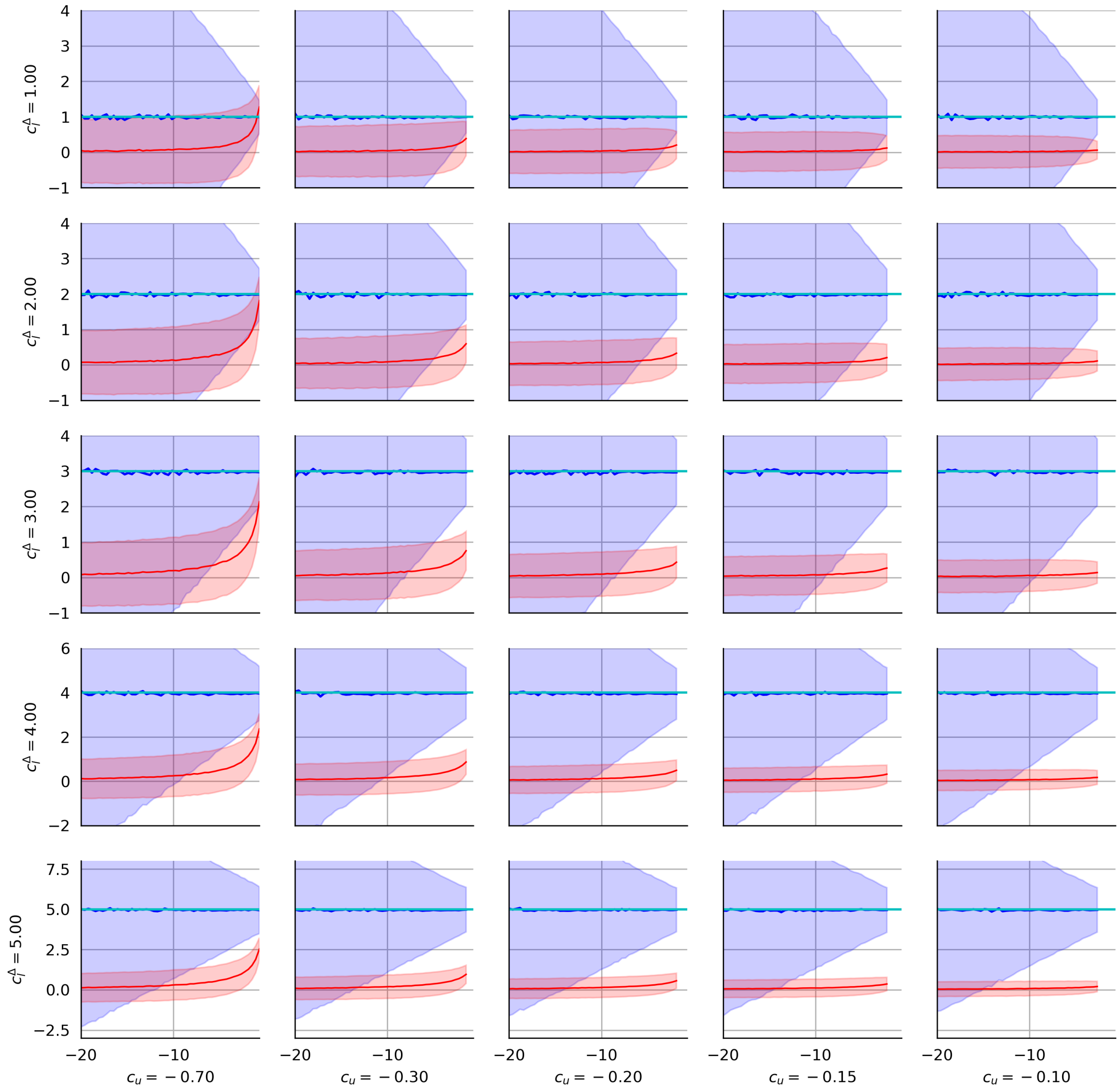

Figure 19. Confidence intervals evaluated with Beta distribution

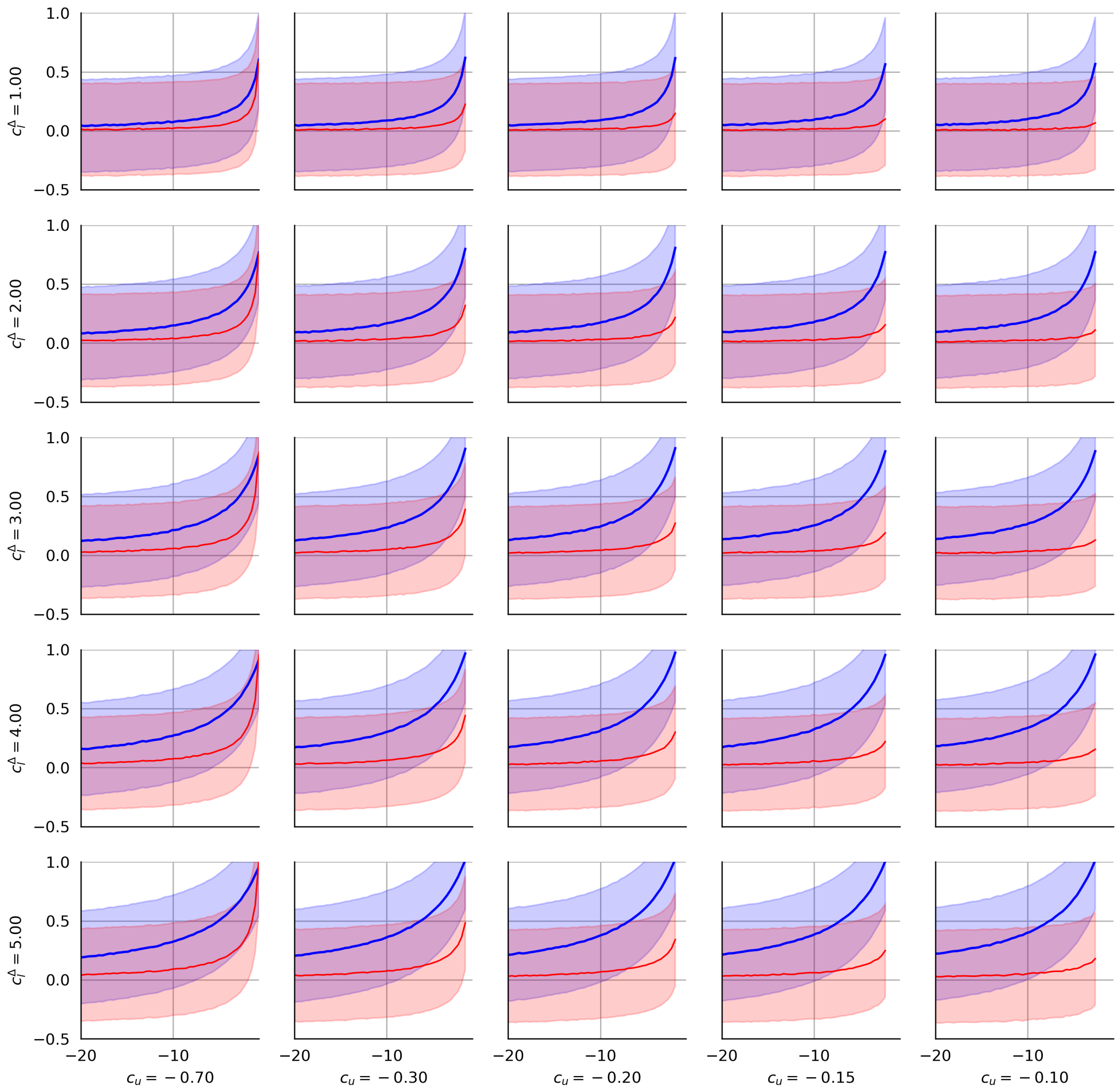

Figure 20. Cohen's d evaluated with Beta distribution

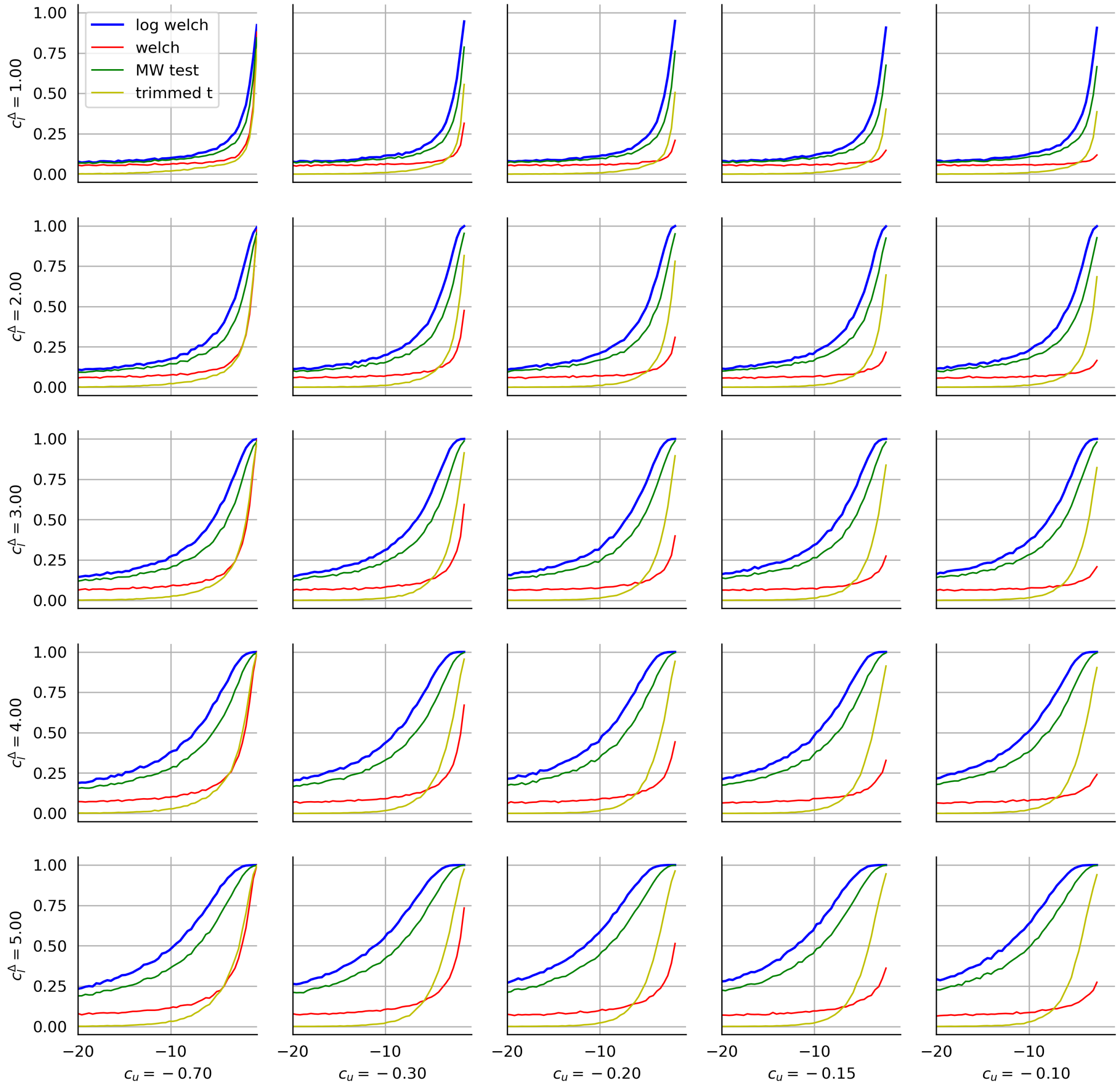

Figure 21. Two-group tests evaluated with Beta distribution

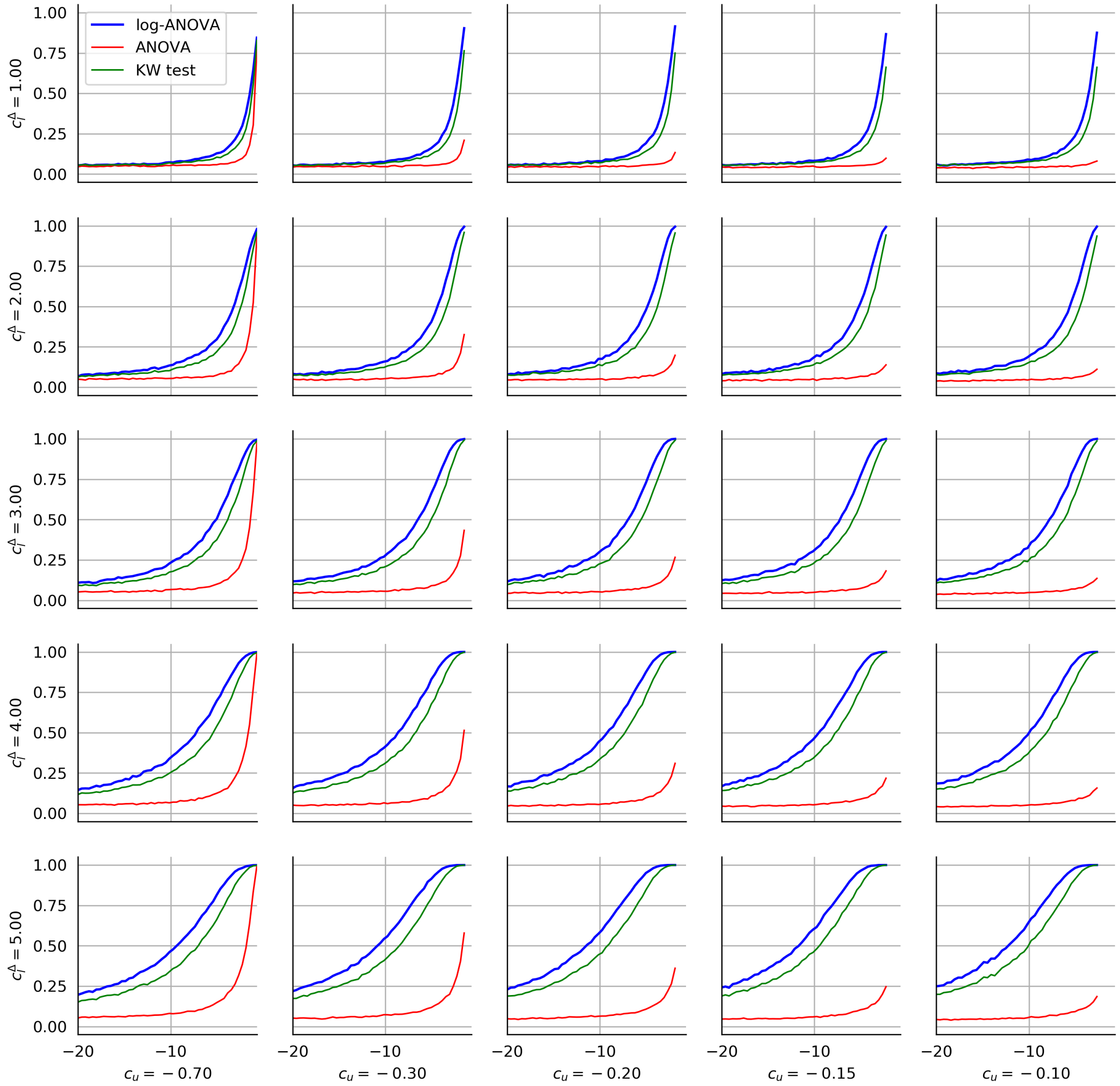

Figure 22. Three-group tests evaluated with Beta distribution

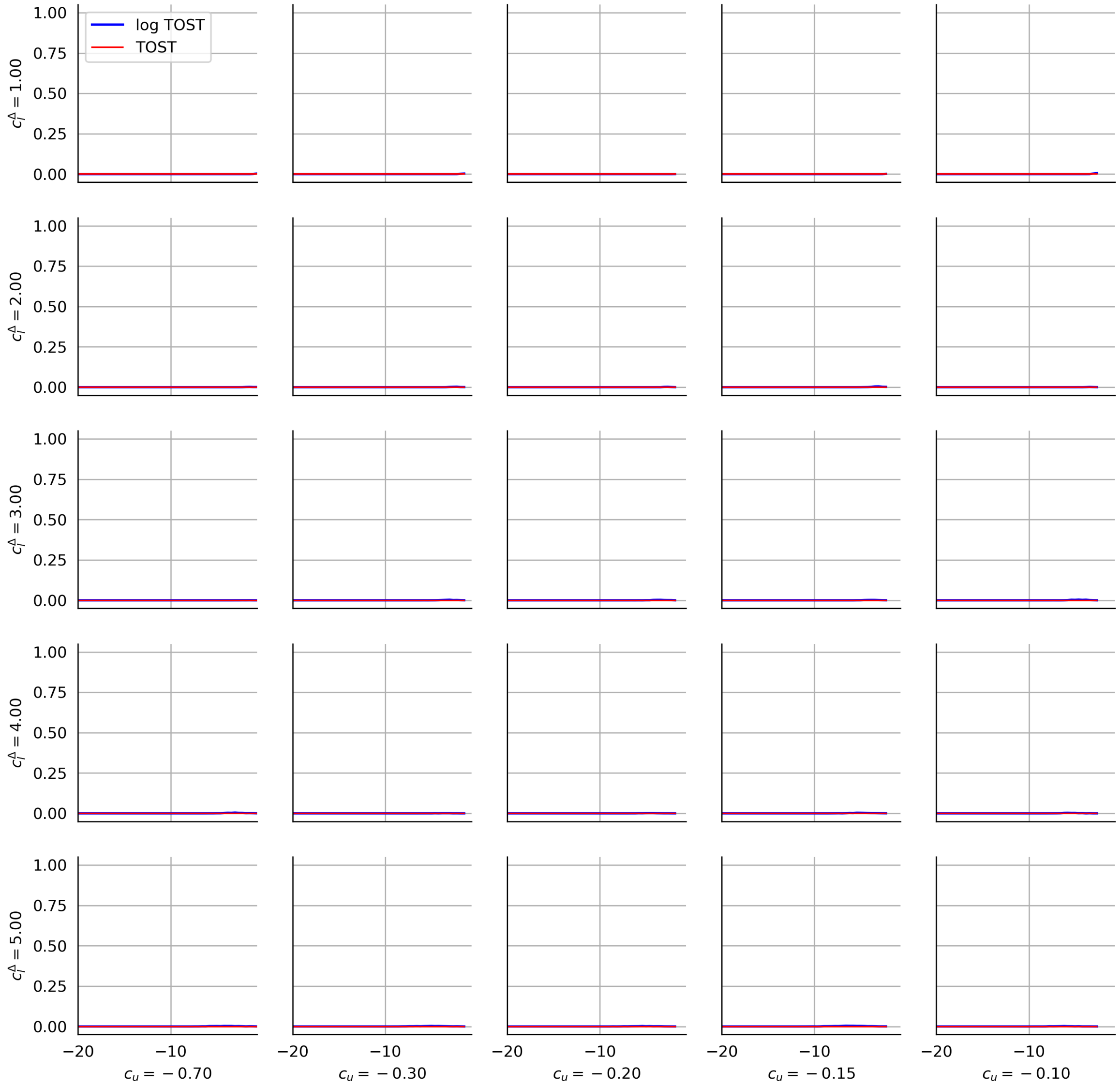

Figure 23. TOST procedure evaluated with Beta distribution

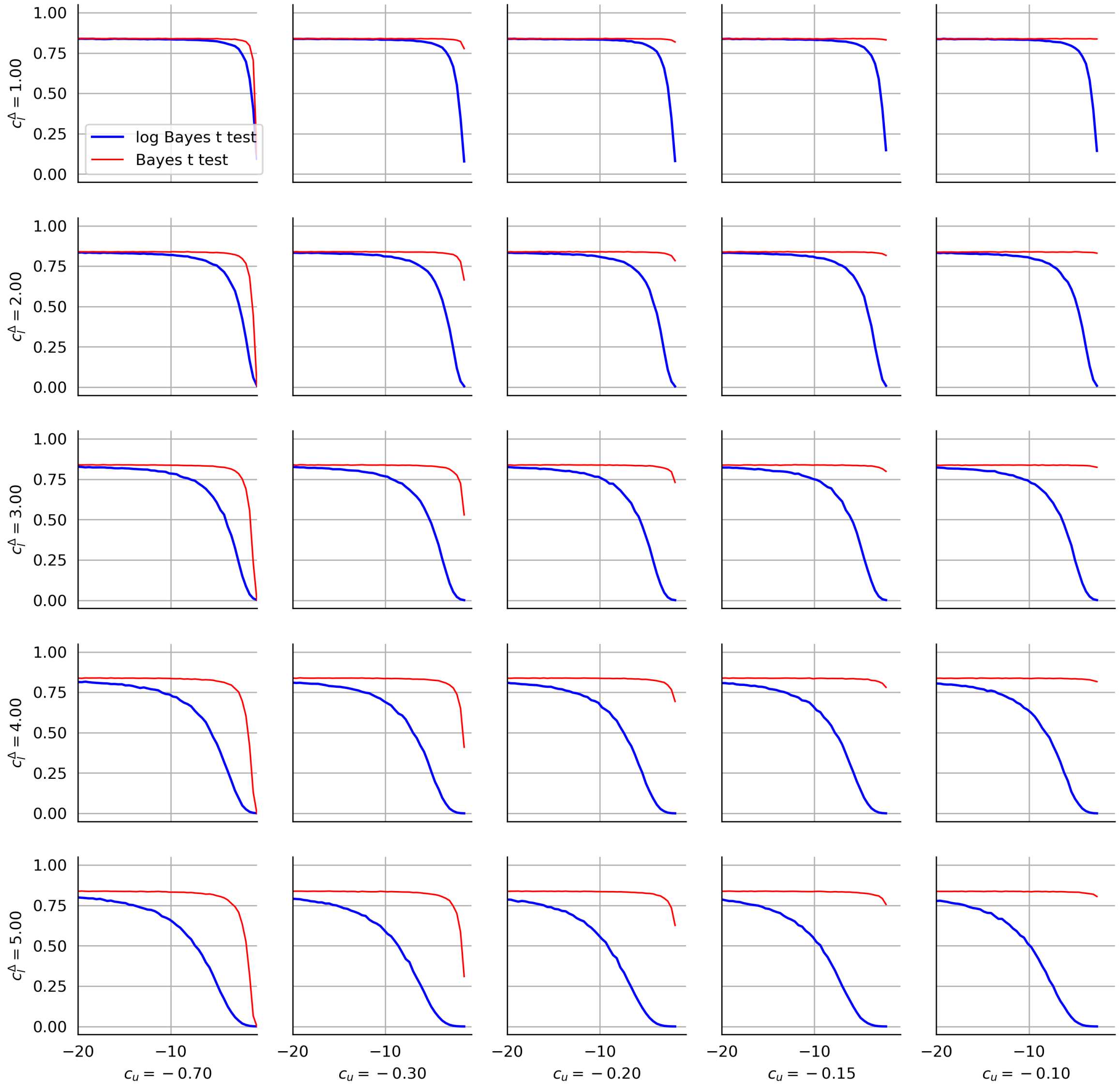

Figure 24. Bayesian t test evaluated with Beta distribution

5 BETA-BINOMIAL DISTRIBUTION

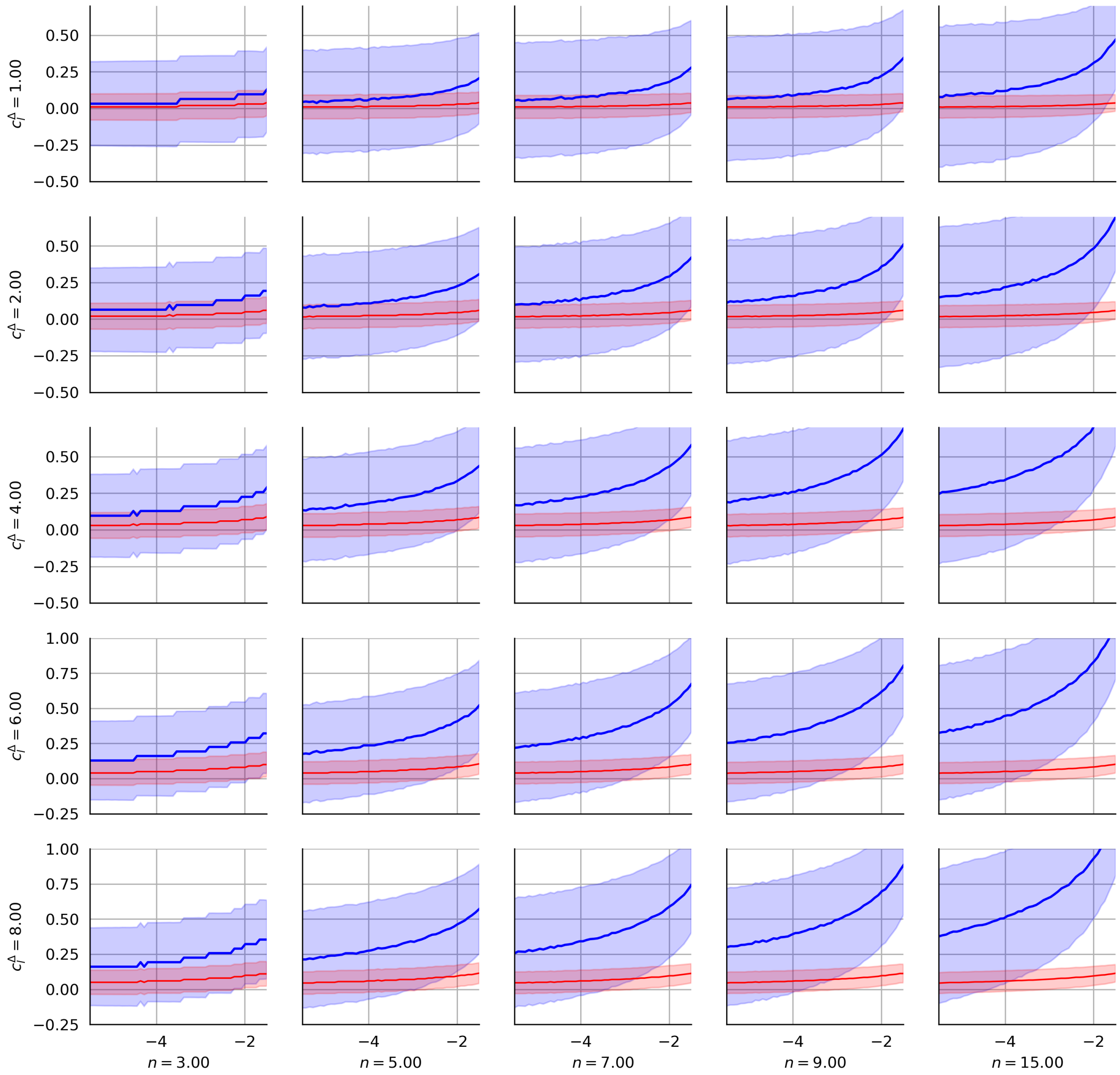

Figure 25. Confidence intervals evaluated with Beta-binomial distribution

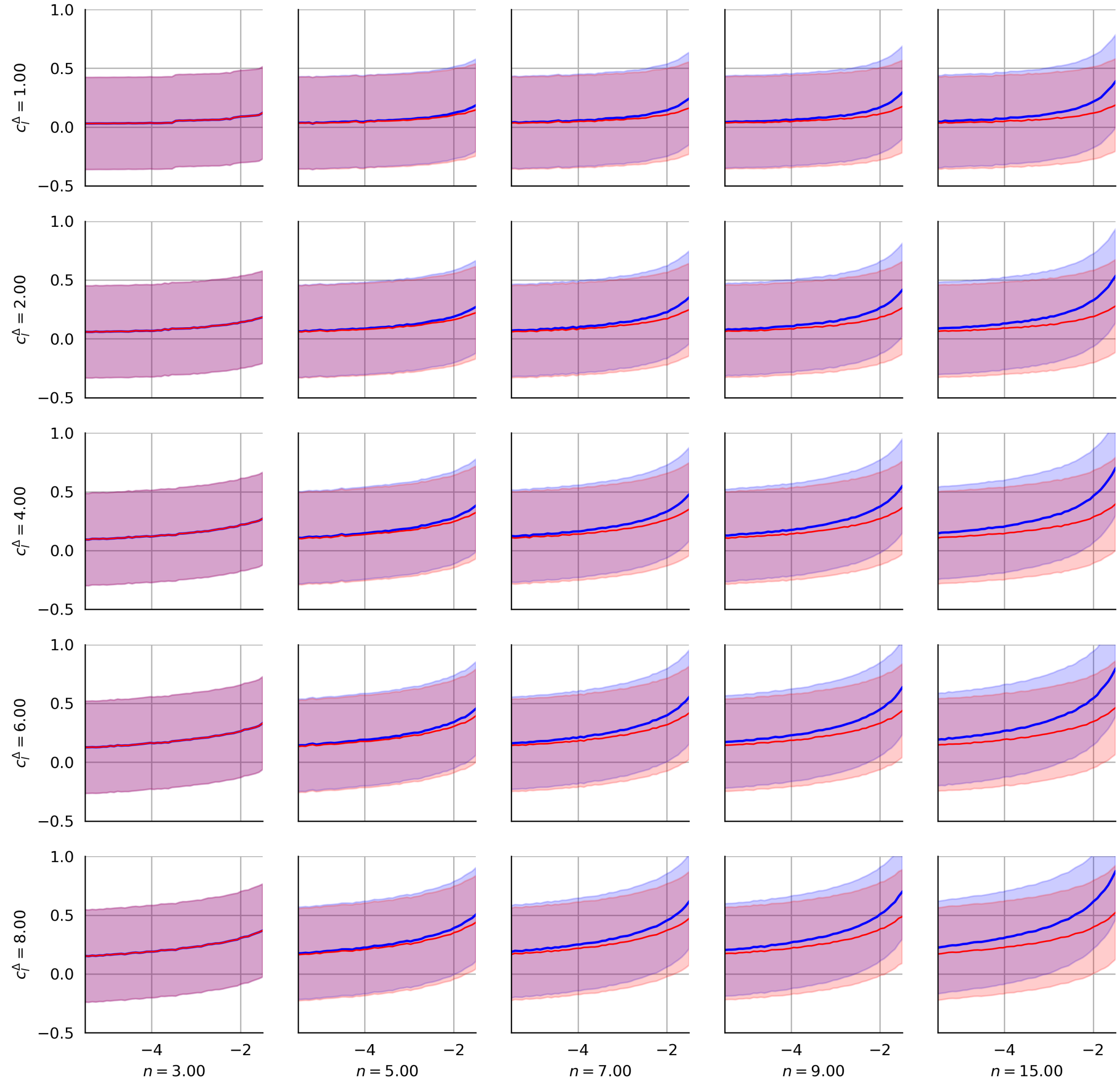

Figure 26. Cohen's d evaluated with Beta-binomial distribution

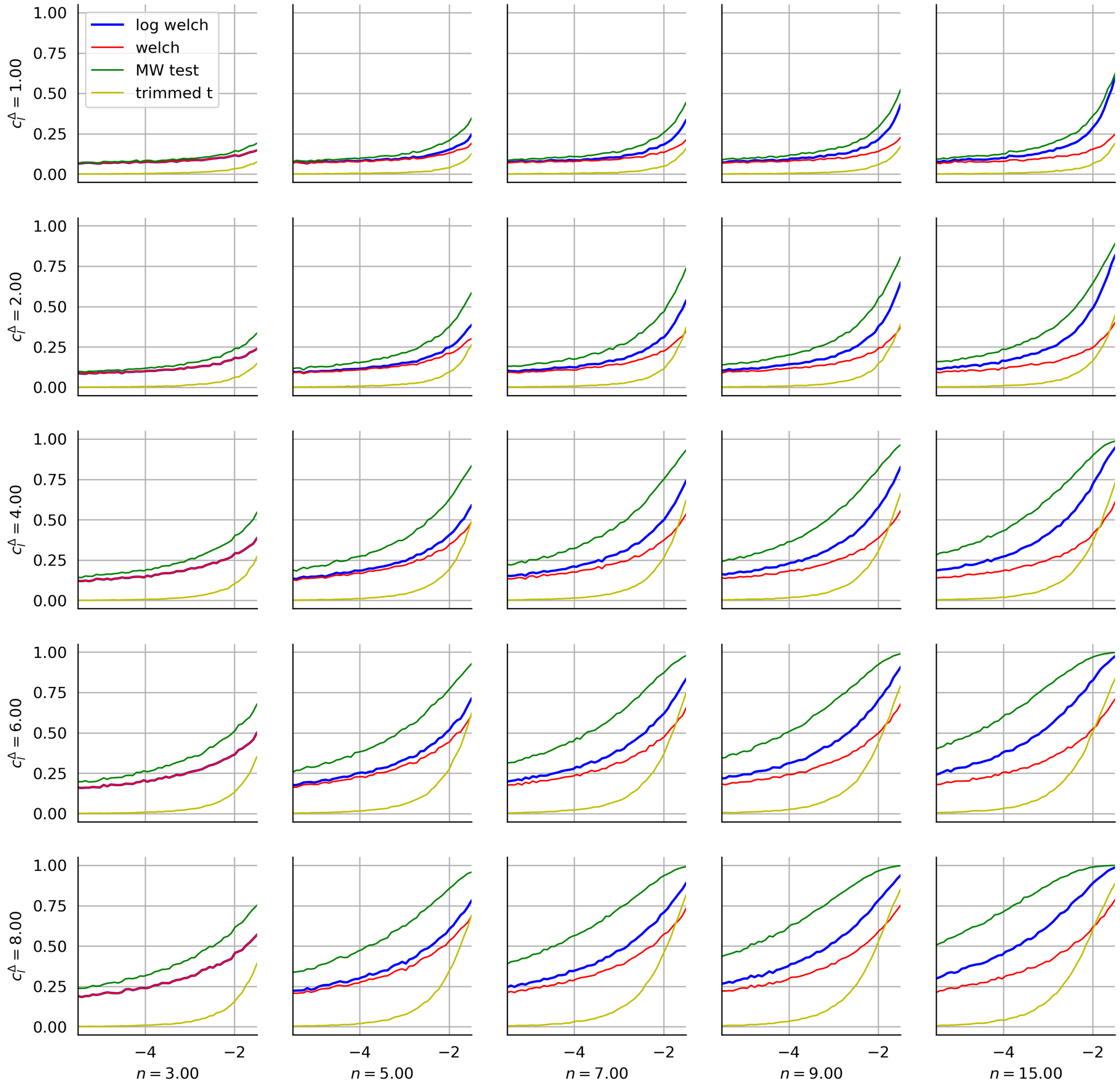

Figure 27. Two-group tests evaluated with Beta-binomial distribution

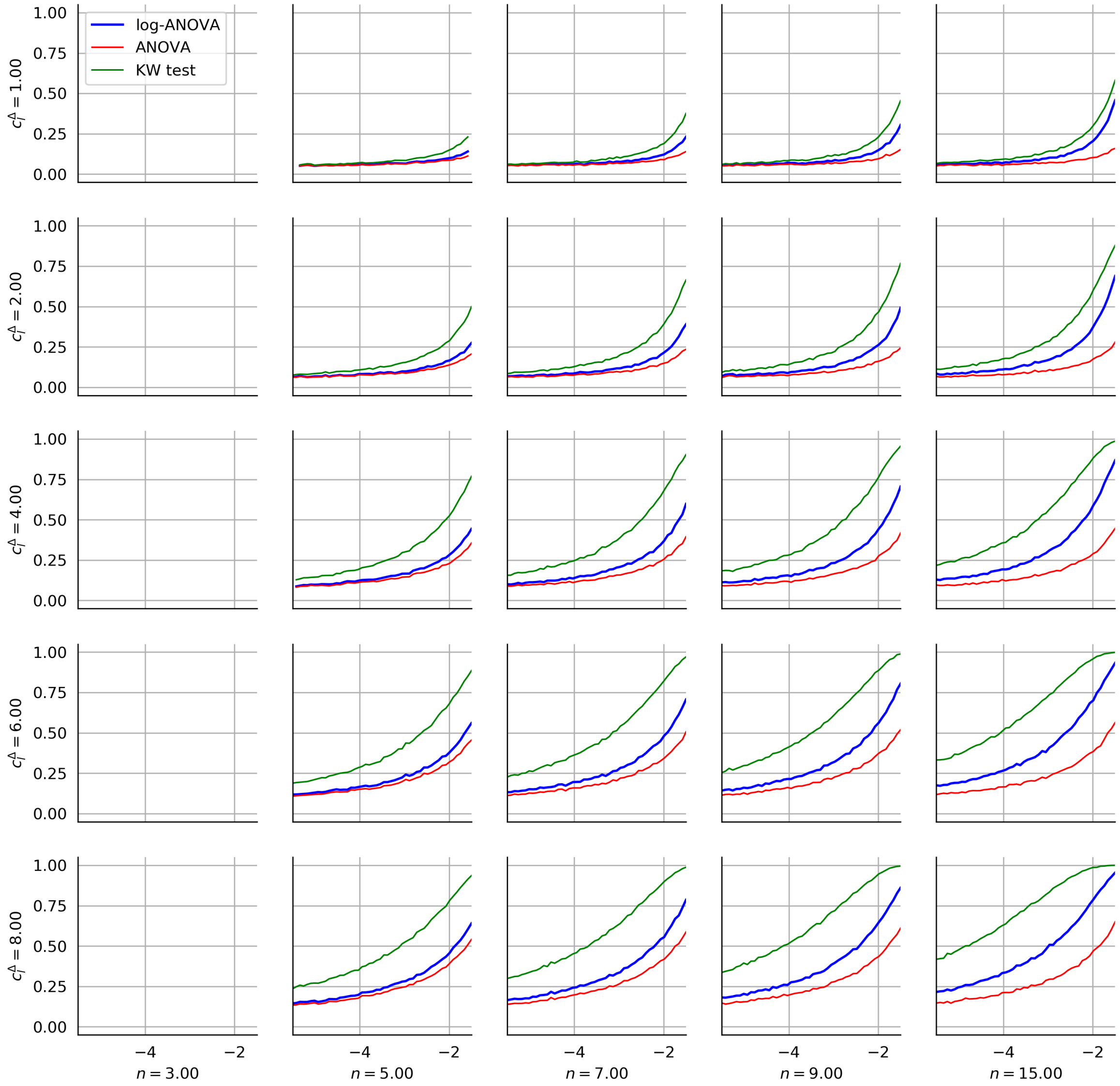

Figure 28. Three-group tests evaluated with Beta-binomial distribution

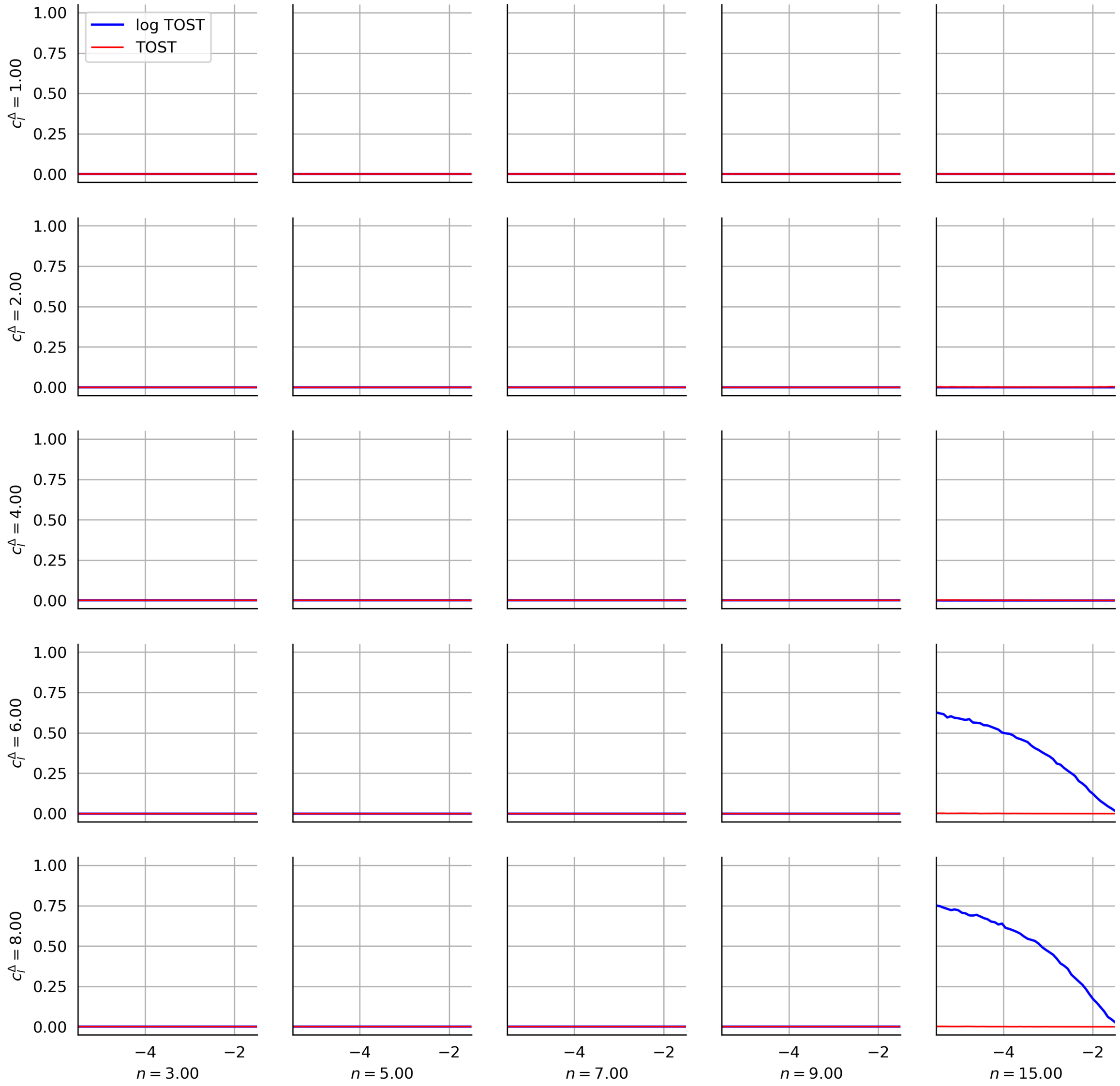

Figure 29. TOST procedure evaluated with Beta-binomial distribution

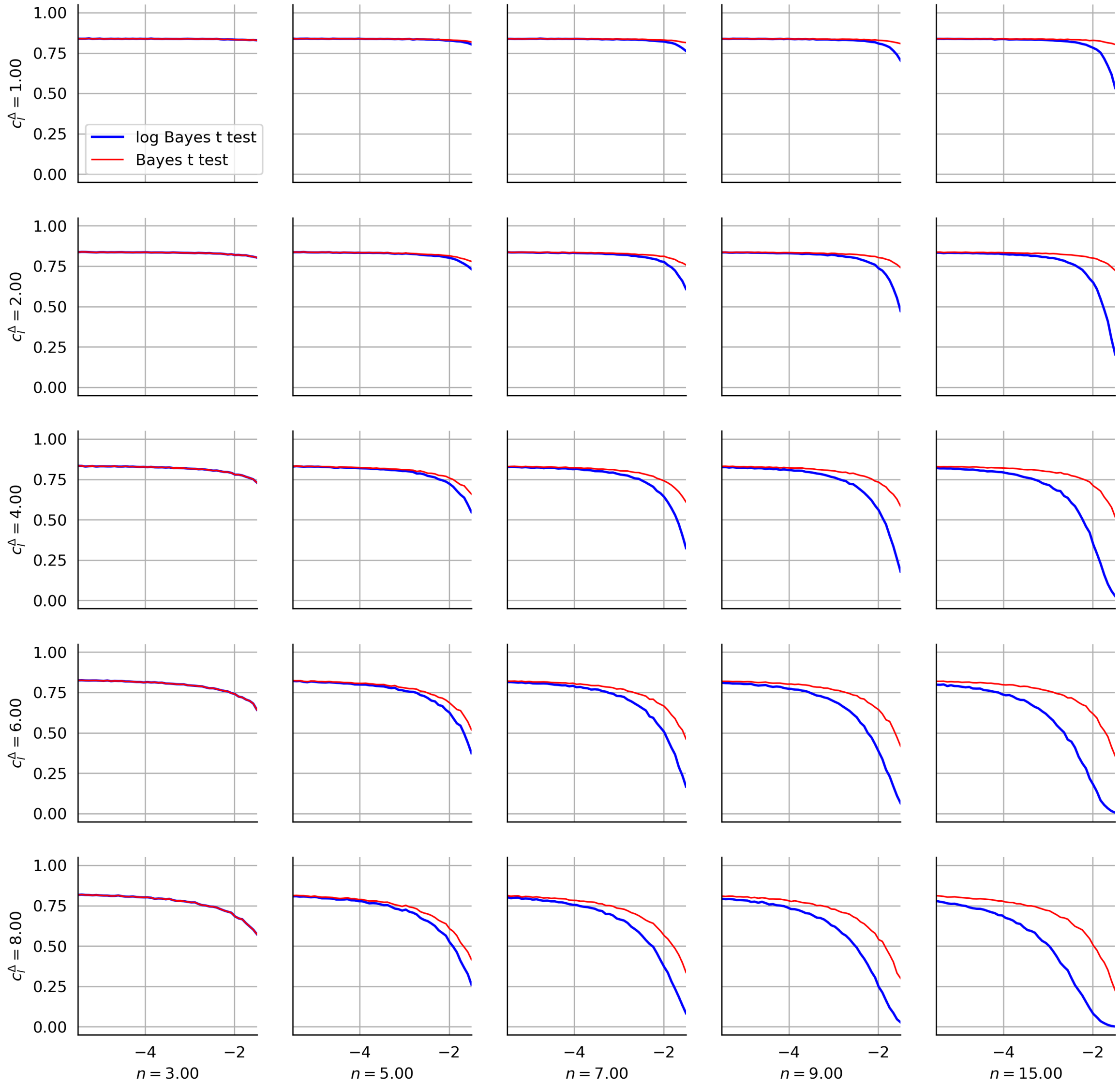

Figure 30. Bayesian t test evaluated with Beta-binomial distribution

6 ORDERED LOGISTIC REGRESSION MODEL

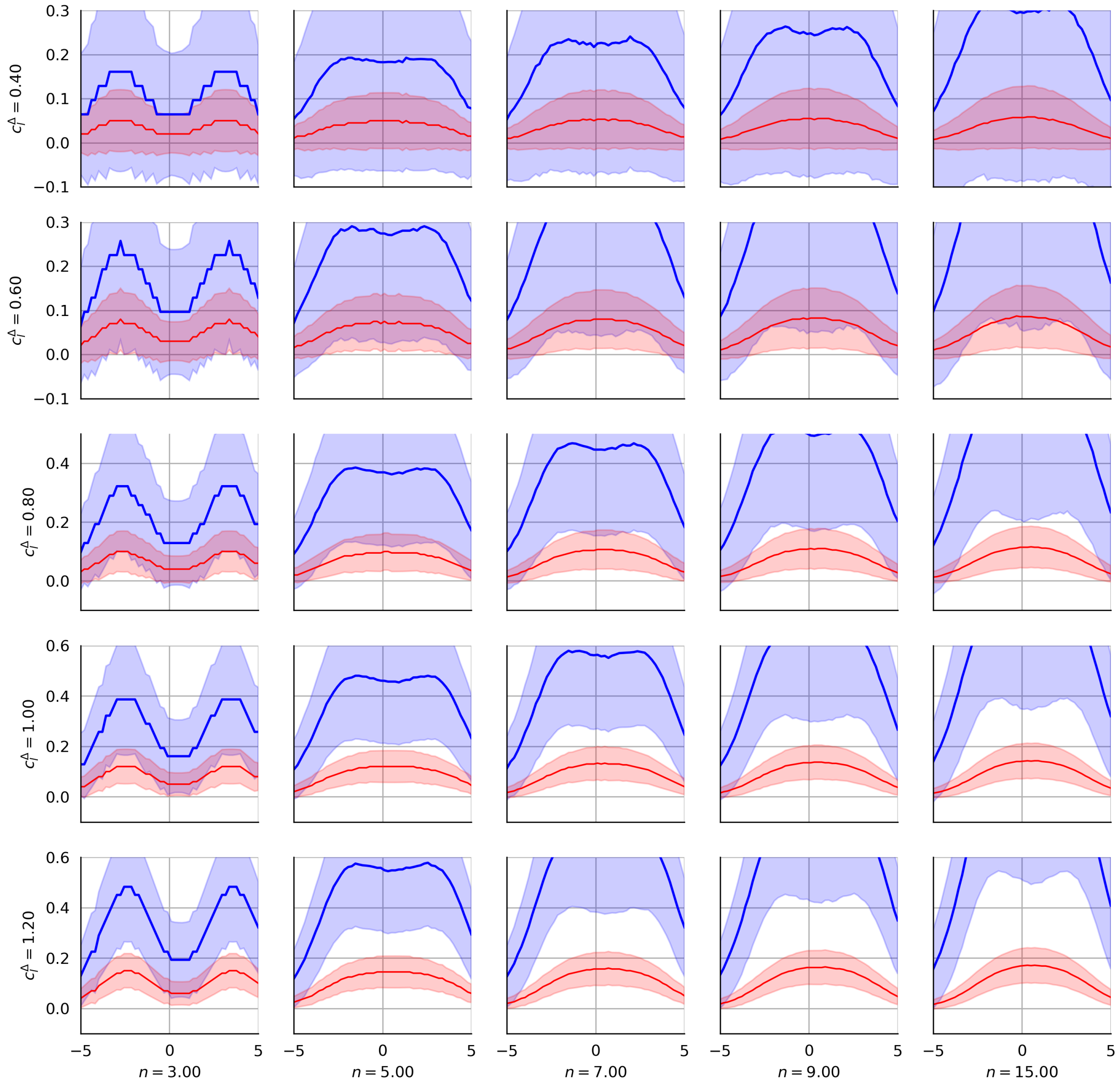

Figure 31. Confidence intervals evaluated with Ordered logistic regression model

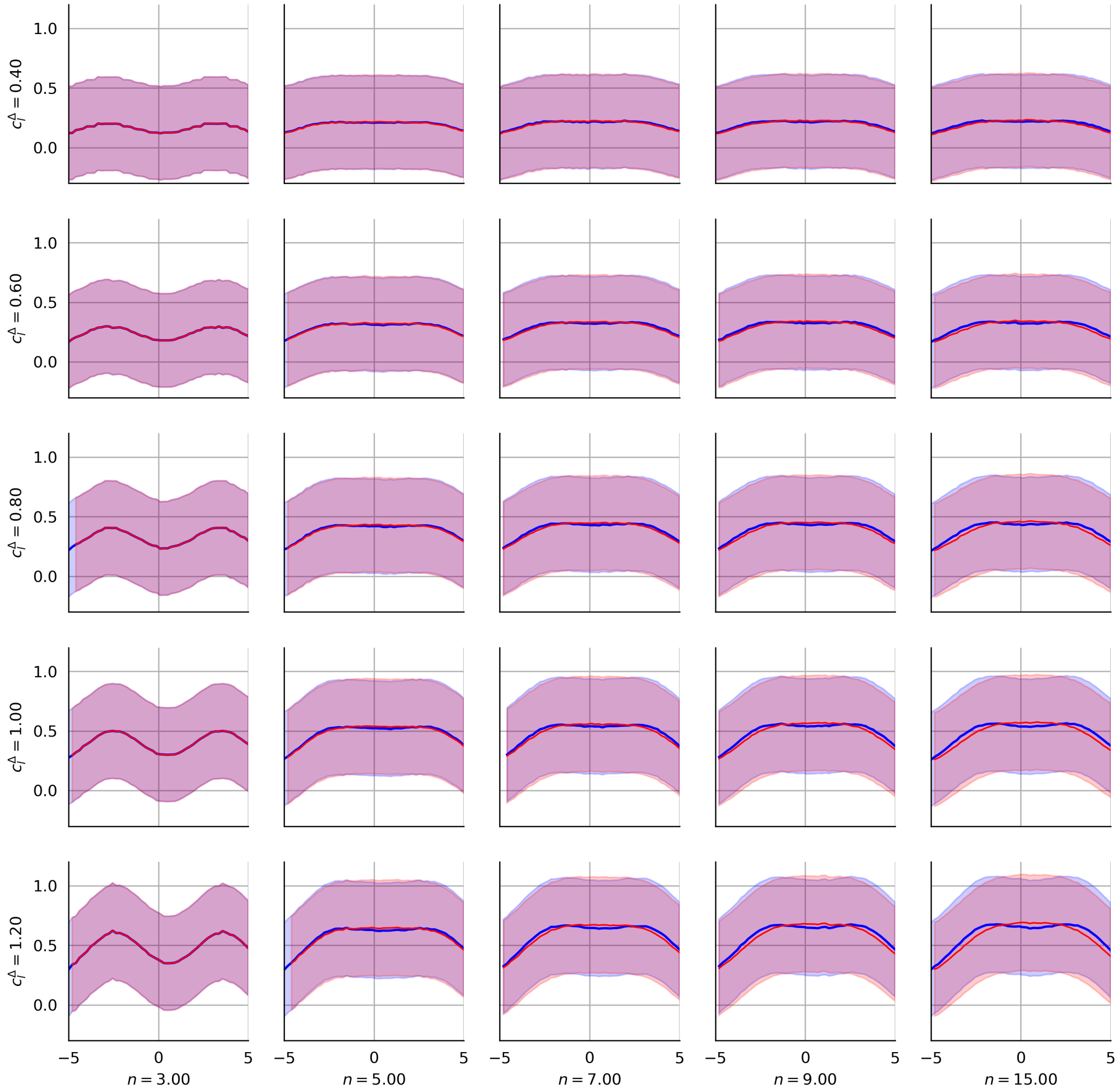

Figure 32. Cohen's d evaluated with Ordered logistic regression model

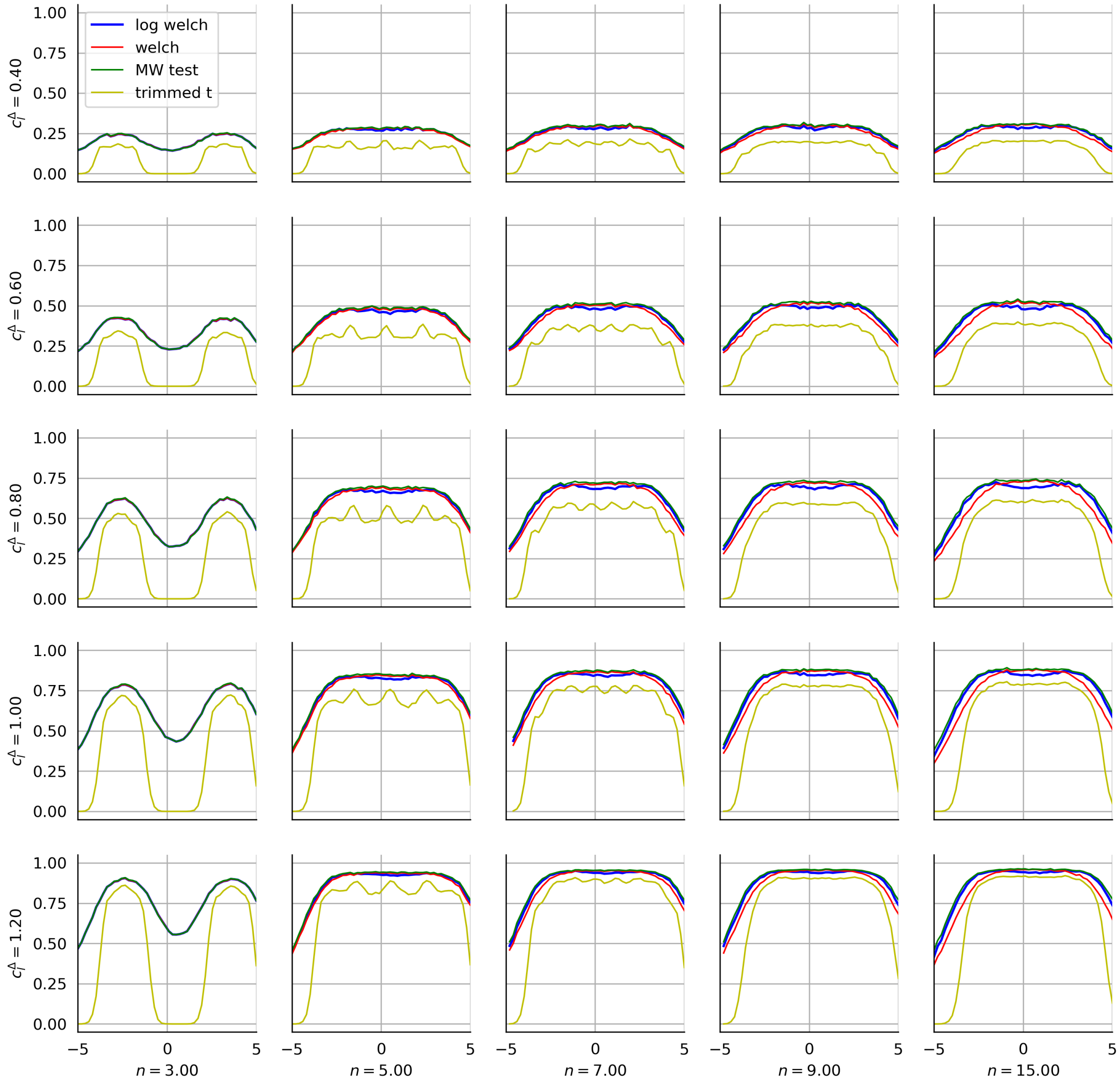

Figure 33. Two-group tests evaluated with Ordered logistic regression model

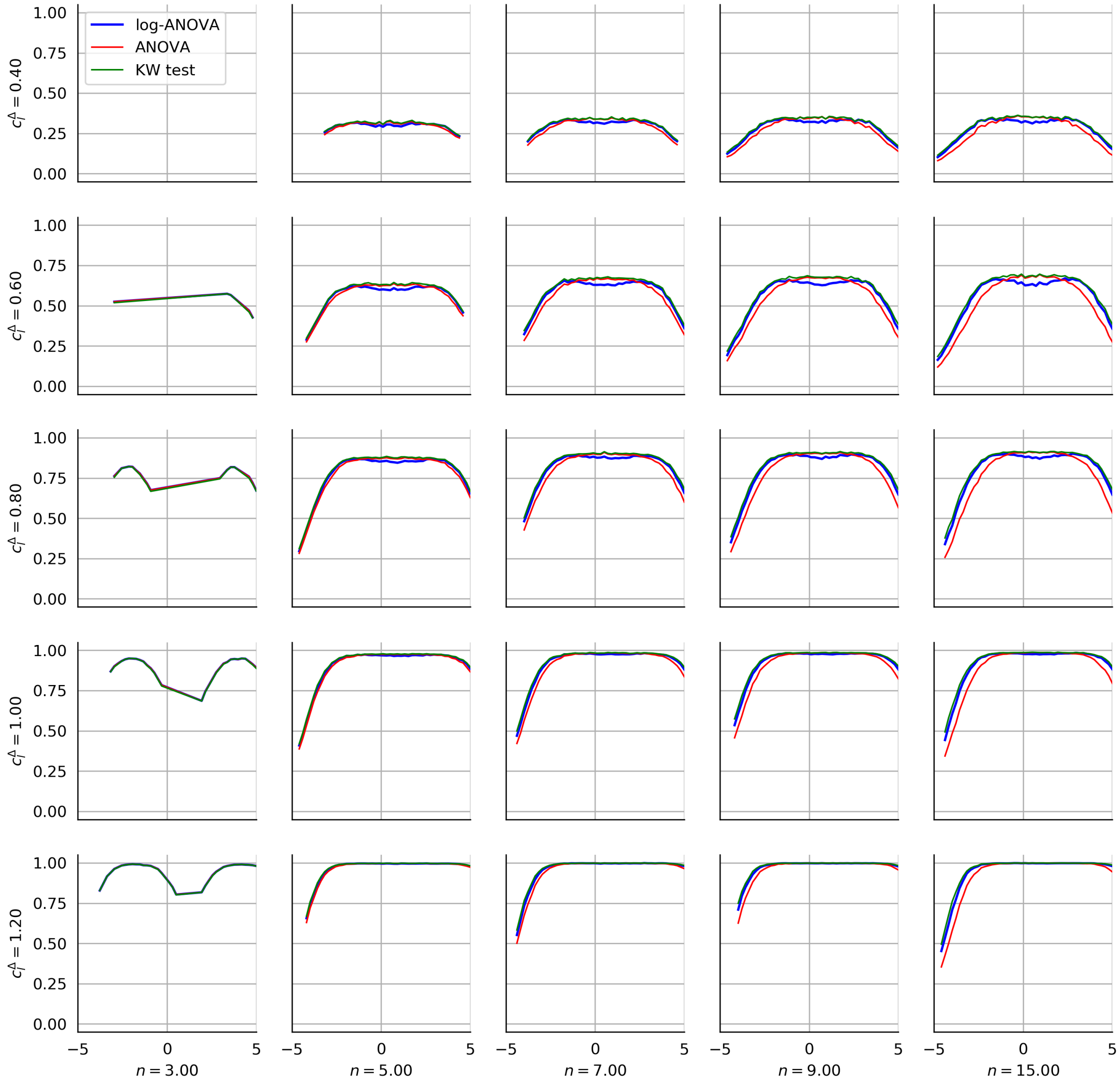

Figure 34. Three-group tests evaluated with Ordered logistic regression model

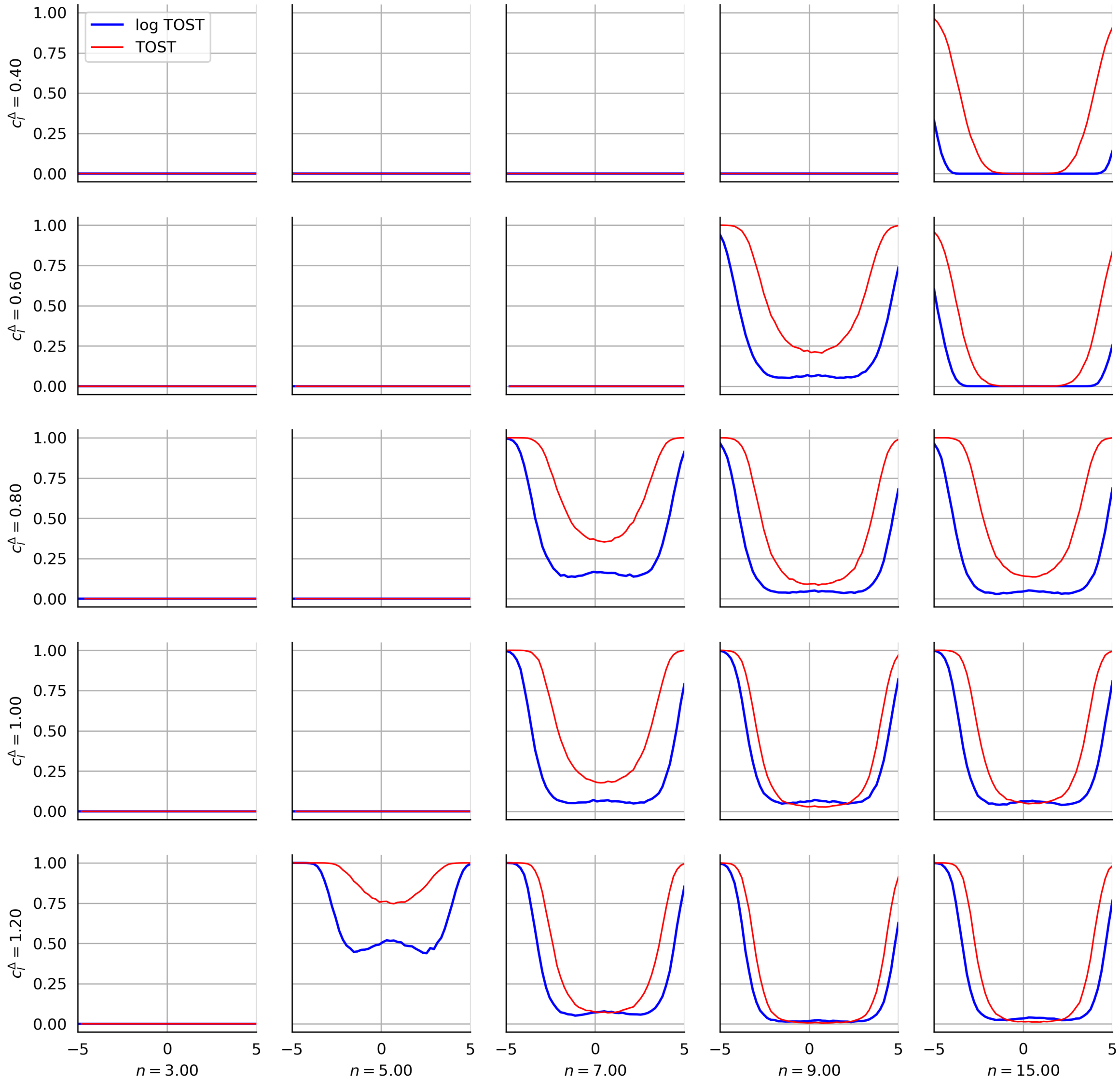

Figure 35. TOST procedure evaluated with Ordered logistic regression model

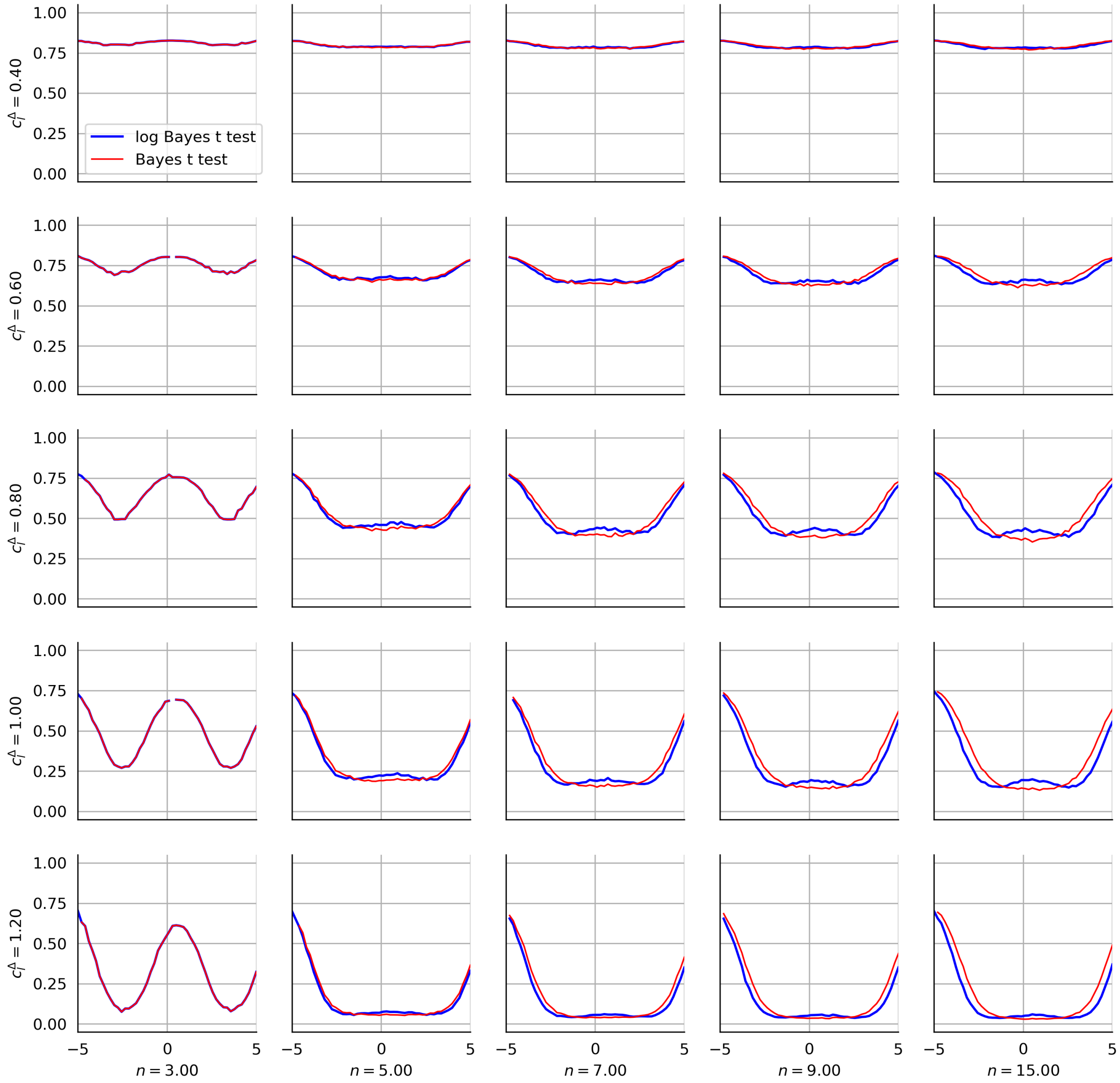

Figure 36. Bayesian t test evaluated with Ordered logistic regression model

7 ANOVA WITH LARGEST C_L^{Δ}

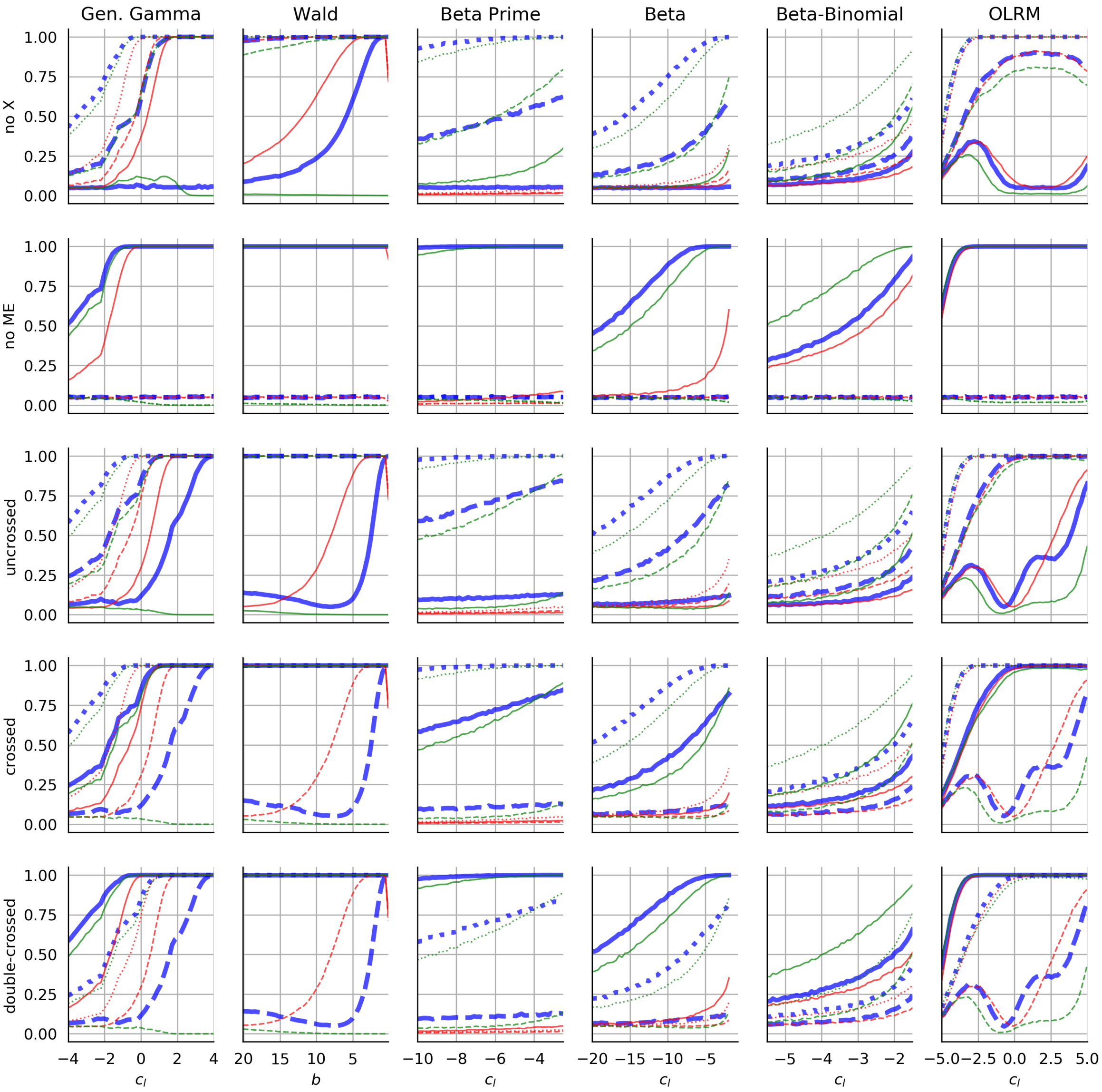

Figure 37. ANOVA with largest c_l^{Δ}. The precise value for each distribution is presented as the upper bound in fourth column in table 2 in the published article.
