# Supplementary material for: Robustness of statistical methods when measure is affected by ceiling and/or floor effect
Source: PLoS One. 2019 Aug 19;14(8):e0220889. doi: 10.1371/journal.pone.0220889 (PMC6699673; doi:10.1371/journal.pone.0220889)
Supplement: Attachment — Submitted filename: SNDwivedi.Plos. One.doc [file pone.0220889.s002.doc]

**Comments on the research article entitles, “Robustness of statistical methods when measure is affected by ceiling and/ or floor effect”**

**This study has addressed a very pertinent topic often useful in applications while dealing with specific data set to answer planned research questions while collecting data. To my understanding, it would have been better if the authors could have shown additional applications of these methods on real data sets.**

**As such, if possible, they need to incorporate this application. Otherwise, if the editor agrees, the article may be accepted for publication.**
